# The global incidence and mortality of contrast-associated acute kidney injury following coronary angiography: a meta-analysis of 1.2 million patients

**DOI:** 10.1007/s40620-021-01021-1

**Published:** 2021-06-02

**Authors:** Zhubin Lun, Liwei Liu, Guanzhong Chen, Ming Ying, Jin Liu, Bo Wang, Jingjing Liang, Yongquan Yang, Shiqun Chen, Yibo He, Edmund Y. M. Chung, Jiyan Chen, Jianfeng Ye, Yong Liu

**Affiliations:** 1 Department of Cardiology, Guangdong Provincial Key Laboratory of Coronary Heart Disease Prevention, Guangdong Cardiovascular Institute, Guangdong Provincial People’s Hospital, Guangdong Academy of Medical Sciences, Guangzhou, 510080 China; 2 Department of Cardiology, Dongguan TCM Hospital, Dongguan, 523000 Guangdong China; 3 The First School of Clinical Medicine, Guangdong Medical University, Zhanjiang, 523808 China; 4 Department of Cardiology, Shunde Hospital of Southern Medical University, Shunde, China; 5 Prince of Wales Hospital, Sydney, Australia; 6 Northern Sydney Clinical School, The University of Sydney, Sydney, Australia

**Keywords:** Contrast-associated acute kidney injury, Coronary angiography, Incidence, Mortality, Meta-analysis

## Abstract

**Background:**

Contrast-associated acute kidney injury (CA-AKI) is a common complication after coronary angiography (CAG), which brings a poor prognosis. But up to now, there were fewer studies to discuss the incidence of CA-AKI comprehensively. We comprehensively explore the incidence of CA-AKI after coronary angiography.

**Methods:**

We searched Medline, Embase, and Cochrane Database of Systematic Reviews (to 30th June 2019). We evaluated the world’s incidence of the CA-AKI, and associated mortality, and to described geographic variations according to countries, regions, and economies. CA-AKI was defined as an increase in serum creatinine ≥ 0.5 mg/dl or ≥ 25% within 72 h. Random effects model meta-analyses and meta-regressions was performed to derive the sources of heterogeneity.

**Results:**

A total of 134 articles (1,211,106 participants) were included in our meta-analysis. Most studies originated from China, Japan, Turkey and United States, from upper middle income and high income countries. The pooled incidence of CA-AKI after coronary angiography was 12.8% (95% CI 11.7–13.9%), and the CA-AKI associated mortality was 20.2% (95% CI 10.7–29.7%). The incidence of CA-AKI and the CA-AKI associated mortality were not declined over time (Incidence rate change: 0.23% 95% CI − 0.050 to 0.510 *p* = 0.617; Mortality rate change: − 1.05% 95% CI − 3.070 to 0.970 *p* = 0.308, respectively).

**Conclusion:**

CA-AKI was a universal complication in many regions, and the burden of CA-AKI remains severe. In clinical practice, physicians should pay more attention to the occurrence and active prevention and treatment of CA-AKI.

**Graphic abstract:**

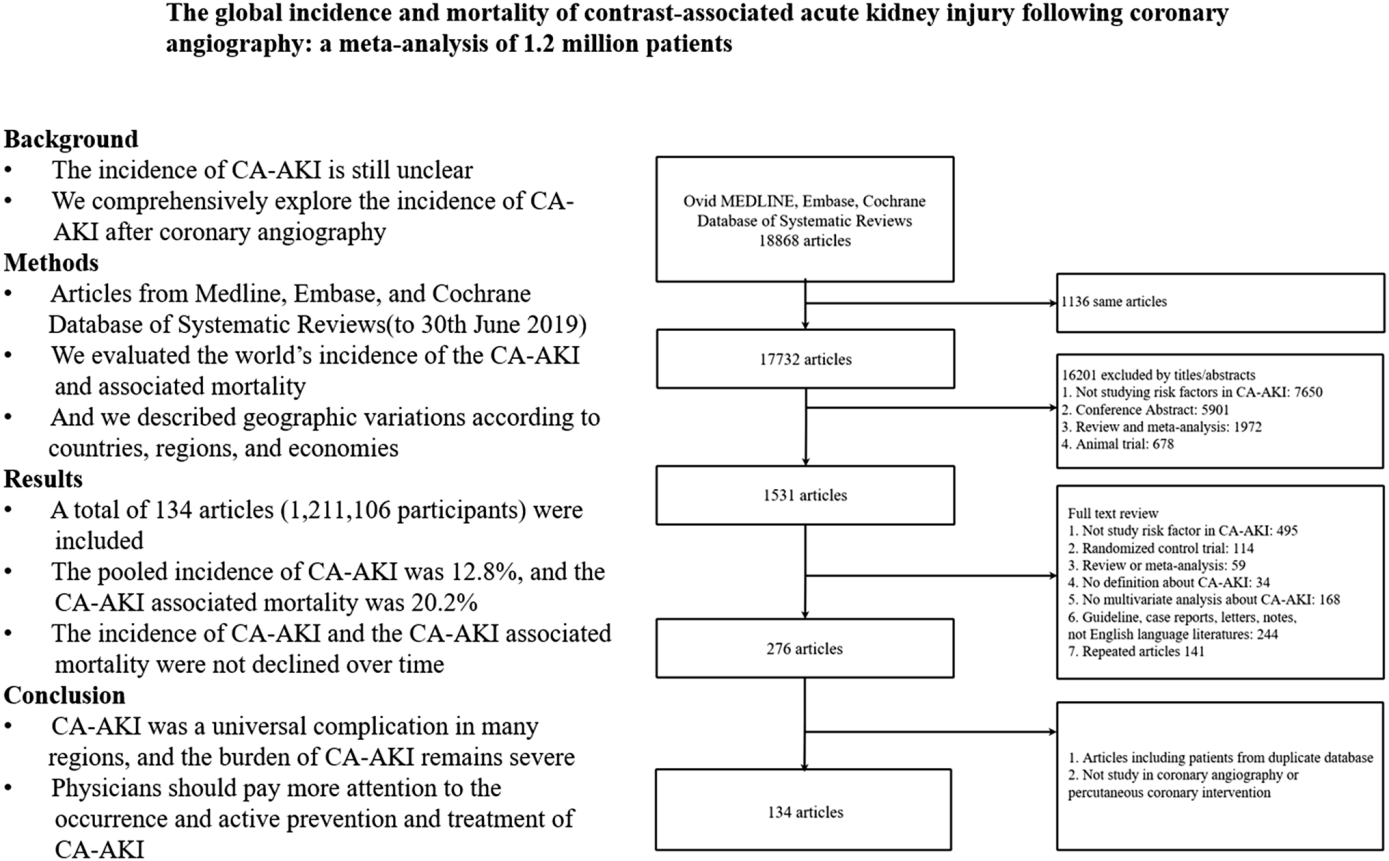

**Supplementary Information:**

The online version contains supplementary material available at 10.1007/s40620-021-01021-1.

## Introduction

As the burden of cardiovascular disease increases, cardiovascular diseases have become the world's leading cause of death, and the number of coronary angiography continues to rise [1]. Contrast-associated acute kidney injury (CA-AKI) is one of the major complications of coronary angiography (CAG), and the occurrence of CA-AKI is related to the toxicity of contrast agent, which leads to kidney function loss, with apoptosis and tubular necrosis [2, 3]. The high osmolality contrast agent and ionic contrast agent were related to the high risk of CA-AKI [4, 5]. At the same time, the risk of CA-AKI increases with each additional 100 ml of contrast agent [6]. In addition, renal ischemic injury caused by vasoactive substances such as endothelin, nitric oxide and prostaglandins may increase the risk of CA-AKI [7–9]. The occurrence of CA-AKI is significantly associated with prolonged hospitalization and with an increase in short and long-term mortality [10, 11].

Numerous studies have suggested that the incidence of CA-AKI ranged from 3 to 50% [12–14]. Chalikias et al. mentioned that the current incidence of CA-AKI was difficult to calculate due to the influence of many factors (CA-AKI definition, prevalence of comorbidity, including diabetes mellitus, pre-existing renal disease, heart failure, anemia) and was also related to clinical setting [15]. Furthermore, it has been confirmed in previous studies that age > 75 years, hypotension, intra-aortic balloon pump and hypoproteinemia are risk factors for CA-AKI [6, 16]. In this context, Hoste suggested that the current incidence of CA-AKI and the prognosis varied, depending on the diagnostic criteria and population characteristics [17]. Up to now, the incidence of CA-AKI around the world has not been systematically reviewed, and further research is needed to determine its stages of severity and associated mortality.

This meta-analysis was conducted to estimate the world incidence and mortality associated with CA-AKI after coronary angiography, and to describe variations according to countries, regions, comorbidities and economic aspects.

## Materials and methods

### Data sources and literature search

A literature search was performed in MEDLINE, Embase and the Cochrane Database with the searching terms “contrast-induced acute kidney injury”, “risk” and “acute kidney injury” (Supplemental Item 1). The search was limited to human studies written in English and published before 30th June, 2019 (PROSPERO register number: CRD42019121534).

### Study selection

Studies were included if they fulfilled the following criteria: (1) studies reporting on the risk factors of CA-AKI and (2) retrospective and prospective observational studies. Studies were excluded if they were classified as (1) duplicate articles; (2) contained no precise definitions of CA-AKI; (3) randomized controlled trials, meta-analyses, case reports, editorials, animal studies. At least two authors independently assessed the citations retrieved in the electronic search and identified eligible studies.

### Data extraction and quality assessment

At least two independent authors (ZB Lun and LW Liu) assessed each study by screening the title, abstract or full-text independently. Then these two reviewers extracted data on the study characteristics, the incidence of CA-AKI and associated potentially modifiable risk factors. Newcastle–Ottawa Scale was performed to evaluate the quality of the included studies. The authors resolved discrepancies in study selection, data extraction and assessment of study quality through discussion with an arbitrator (Y Liu).

### Definitions and outcomes

According to the geographical scheme designed by the United Nations Statistics Division, countries are classified into different continents and world regions [18]. Countries’ economies were assessed according to three ranges of gross national income per capita derived from the World Bank’s classification of income of economies: lower middle, upper middle, and high income countries [19, 20]. Using the World Health Organization’s World Health Statistics data, countries are also classified according to the percentage of total national health expenditure to gross domestic product (GDP) [21].

We evaluated the incidence of CA-AKI by three definitions. The definition of CA-AKI was an increase in serum creatinine ≥ 0.5 mg/dl or ≥ 25% from baseline within 72 h after exposure to contrast. In addition, we also analyzed the incidence of two other definitions of CA-AKI, as reported in Supplementary material. The two definitions of CA-AKI were: an increase in serum creatinine ≥ 0.3 mg/dl or ≥ 50% from baseline within 72 h and the criteria of acute kidney injury network (AKI stage 1, ≥ 0.3 mg/dl absolute or 1.5 to 2.0-fold relative increase in serum creatinine; AKI stage 2, > 2- to threefold increase in serum creatinine; AKI stage 3, > threefold increase in serum creatinine or serum creatinine > 4.0 mg/dl with an acute increase of > 0.5 mg/dl) or RIFLE criteria (risk, injury, failure, loss of kidney function and end-stage kidney disease) [22] or Kidney-disease-improving-global outcomes[23]. The CA-AKI associated mortality was considered as CA-AKI associated all-cause mortality.

### Statistics analysis

The pooled incidence rates of CA-AKI and mortality were assessed with random-effects or fixed-effects models, based on the heterogeneity of included studies. Heterogeneity was quantified by the *Q* statistic and *I*^2^ statistic, which describes the percentage of total variation across studies due to heterogeneity and not due to sampling error [24]. If *I*^2^ was > 50%, a random-effects model was used. Otherwise, the fixed-effects model was adopted [25]. Subgroup analyses and meta-regression were performed for the following subsets of studies: percutaneous coronary intervention related studies, CAG or percutaneous coronary intervention related studies, clinical comorbidities, CA-AKI definitions, countries, continents, latitude, countries income classification, countries total wealth. Funnel plot was used to assess publication bias [26]. Publication bias was considered significant when *p* < 0.05. All analyses were performed with STATA (version 13.0) and R software (version 3.6.1; R Core Team, Vienna, Austria).

## Results

### Study characteristics and quality assessment

**Fig. 1:**
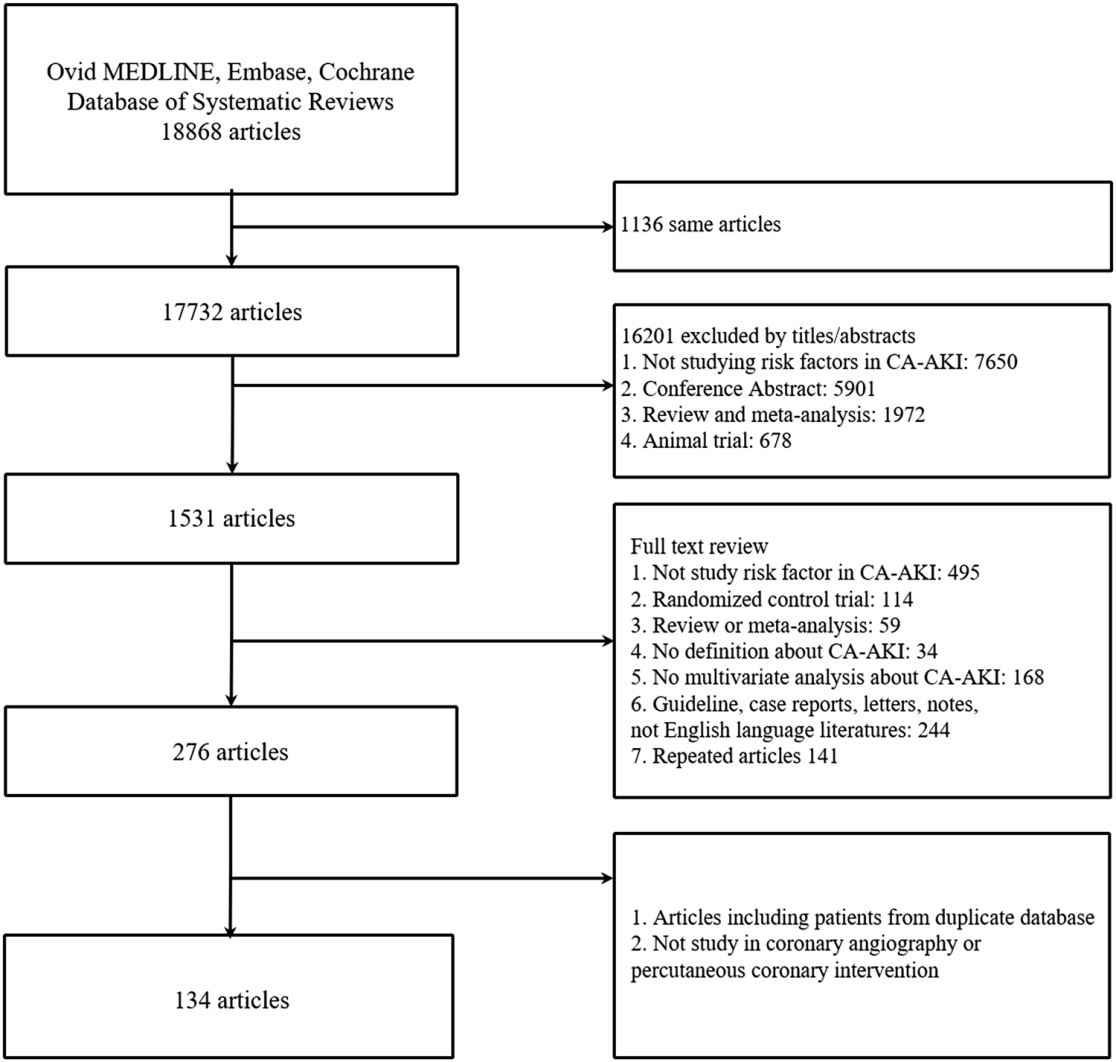
Literature search and selection

A total of 18,868 potentially relevant citations were identified and screened; 17,732 articles were retrieved for detailed evaluation, 134 of which fulfilled eligibility criteria (Supplemental References), representing 1.2 million patients from 24 countries worldwide (Fig. 1). Characteristics of all 134 studies are displayed in Supplemental Table 1. All studies were published in English and publication spanned 17 years. In the Table 1, most studies (23.2%) originated from Turkey (23 studies), followed by China (20 studies), United States (15 studies), Japan (10 studies), Italy (9 studies), Korea (7 studies). The top-three world zones where studies were conducted were Asia (67 studies), North America (15 studies), and Europe (14 studies). Most studies (48.5%) originated from high-income countries (48 studies) followed by upper middle income countries (47 studies). Most studies also originated from countries that spent over 5% of GDP on total health expenditure (94 studies). There were 97 studies (98.0%) that originated from countries located north of the equator. All studies were considered high-quality studies. Among them, 78 studies got 9 points, 45 studies got 8 points, and the rest got 7 points (Supplement Table 2).

**Table 1:** Pooled incidence of CA-AKI

Subgroup	Studies (*n*)	Patients (*n*)	With CA-AKI (*n*)	CA-AKI incidence rate (%)	95% confidence interval	Test for heterogeneity	
						*I*^2^ Index	*Q* text *p* Value
All studies	99	171,645	13,607	12.8	11.7–13.9	98.7	< 0.001
Studies by countries							
China	29	110,878	7159	12.4	9.9–14.9	97.7	< 0.001
Japan	14	14,508	1635	14.5	10.2–18.7	95.1	< 0.001
Germany	1	458	125	27.3	23.2–31.4	–	–
India	3	1806	171	12.4	6.9–18.0	92.1	< 0.001
Italy	9	8943	922	12.9	10.3–15.5	92.9	< 0.001
Korea	7	4530	463	10.0	6.9–13.1	92.8	< 0.001
Turkey	23	17,945	2571	14.7	12.2–17.1	95.9	< 0.001
United States	15	104,621	5703	10.4	8.2–12.5	99.5	< 0.001
Brazil	1	201	48	23.9	18.0–29.8	–	–
Iran	2	505	101	19.8	5.8–33.8	94	< 0.001
Studies by continent							
Asia	67	55,247	6405	13.2	11.9–14.6	96.7	< 0.001
Africa	1	200	43	21.5	15.8–27.2	–	–
Europe	14	11,149	1392	12.7	10.1–15.3	94.6	< 0.001
North America	15	104,261	5703	10.4	8.2–12.5	99.5	< 0.001
South America	1	201	48	23.9	18.0–29.8	–	–
Studies by latitude							
North	97	171,217	13,543	12.8	11.7–13.8	98.7	< 0.001
South	2	428	64	15.3	− 1.2–31.8	95.8	< 0.001
Country income classification^#^							
Lower middle income	4	2006	214	15.1	9.2–21.0	93.2	< 0.001
Upper middle income	47	41,143	4956	14.1	12.3–15.9	97.3	< 0.001
High income	49	128,496	8437	11.4	10.0–12.8	99.0	< 0.001
Country total health expenditure (% of GDP)^&^							
< 5	5	5090	429	8.9	6.3–11.4	86.6	< 0.001
5–10	68	56,721	6,735	13.1	11.8–14.5	96.7	< 0.001
> 10	26	109,834	6443	12.2	10.5–14.0	99.3	< 0.001
Studies of percutaneous coronary intervention^$^	63	140,442	10,798	13.3	11.9–14.6	99.0	< 0.001
Studies by clinical setting							
Chronic kidney disease	5	3062	401	14.4	10.6–18.2	84.6	< 0.001
Diabetes mellitus	4	2050	241	12.0	7.6–16.4	89.1	< 0.001
ST-elevation myocardial infarction^^^	23	23,321	2551	12.8	10.3–15.2	97.6	< 0.001

CA-AKI: increase in serum creatinine ≥ 0.5 mg/dl or ≥ 25% from baseline within 72 h after exposure to contrast

^#^According to the World Bank’s classification of income of countries

^&^According to the World Health Organization

^$^Percutaneous coronary intervention: performed from the transfemoral approach according to standard clinical practice

^^^ST-elevation myocardial infarction: defined as ST-segment elevation > 0.1 mV in two consecutively standard leads or > 0.2 mV in two consecutively precordial leads and typical angina > 20 min or new left bundle branch block; Or prolonged (> 30 min) typical chest pain at rest; (2) new ST-segment elevation at the J point in two contiguous leads with the cutoff points: 0.1 mV in all leads other than leads V2–V3 where the following cutoff points applied: 0.2 mV in men 40 years, 0.25 mV in men < 40 years, or 0.15 mV in women or new-onset left bundle branch block; and (3) increased serum biomarkers of myocardial damage; or no report in the original article

### Data synthesis

#### Pooled incidence rate of CA-AKI

**Fig. 2:**
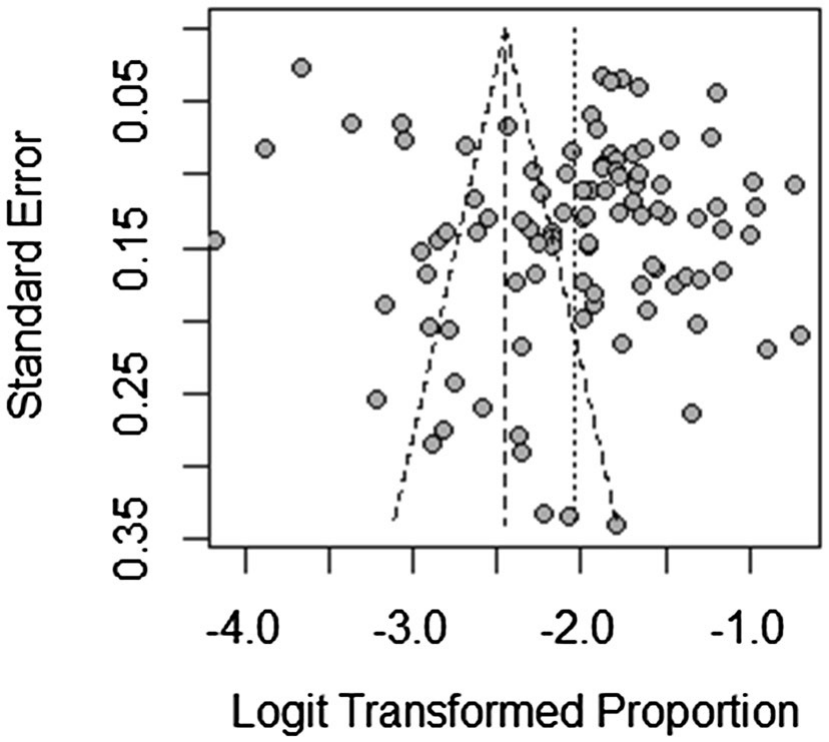
Pooled incidence rate and mortality of CA-AKI by world zones. CA-AKI: increase in serum creatinine ≥ 0.5 mg/dl or ≥ 25% from baseline within 72 h after exposure to contrast

We pooled the incidence rate of CA-AKI by 99 studies, the pooled rate of CA-AKI was 12.8% (95% CI 11.7–13.9%). In addition, the pooled incidence rate of CA-AKI in patients with percutaneous coronary intervention was 13.3% (95% CI 11.9–14.6%). When we study by clinical setting, the incidence rate in patients with chronic kidney disease, diabetes mellitus and ST-elevation myocardial infarction were 14.4%, 12.0% and 12.8%, respectively (Table 1). No publication bias was found, as confirmed by Egger’s tests (*p* = 0.07), and the funnel plot was shown in Fig. 2. As Table 2 knows, the pooled incidence rate of CA-AKI will not change with the growth of the year (rate change: 0.23%, 95% CI − 0.050 to 0.510, *p* = 0.103). At the same time, we found that compared with the incidence from 2002 to 2013, the incidence of CA-AKI from 2014 to 2019 did not increase (rate change: 0.08%, 95% CI − 0.1.490 to 3.070, *p* = 0.497).

**Table 2:** Meta-regression analysis examining the association of CA-AKI incidence rate and its associated mortality rate

Characteristic	Rate change (%)	95% confidence interval	*p* Value
CA-AKI incidence rate			
Year of study publication (per year↑)	0.23	− 0.050 to 0.510	0.103
CA-AKI incidence rate			
Year of study publication (2002–2013) Year of study publication (2014–2019)	Reference 0.08	Reference − 1.490 to 3.070	Reference 0.497
CA-AKI associated mortality rate			
Year of study publication (per year↑)	− 1.05	− 3.070 to 0.970	0.308
CA-AKI associated mortality rate			
Year of study publication (2002–2013) Year of study publication (2014–2019)	Reference − 10.2	Reference − 27.710 to 7.340	Reference 0.255

CA-AKI: increase in serum creatinine ≥ 0.5 mg/dl or ≥ 25% from baseline within 72 h after exposure to contrast

The pooled incidence rate of other definitions of CA-AKI were 15.1% (95% CI 12.9–17.4%) and 16.0% (95% CI 12.6–19.5%), respectively (detailed in Supplementary Table 3).

#### Pooled CA-AKI Associated Mortality Rate

As shown in Table 3, the pooled CA-AKI associated mortality rate was 20.2% (95% CI 10.7–29.7%). As Table 2 knows, the pooled CA-AKI associated mortality rate will not change with the growth of the year (rate change: − 1.05%, 95% CI − 3.070 to 0.970, *p* = 0.308). Compared with the pooled CA-AKI associated mortality rate from 2002 to 2013, the mortality rate of CA-AKI from 2014 to 2019 did not increase (rate change: − 10.2%, 95% CI − 0.27.710 to 7.340, *p* = 0.497).

**Table 3:** Pooled CA-AKI associated mortality rate

Subgroup	Studies (*n*)	Patients (*n*)	With CA-AKI (*n*)	Deaths (*n*)	Pooled mortality rate (%)	95% confidence interval	Test for heterogeneity	
							*I*^2^ Index	*Q* text *p* Value
All studies	19	31,298	3149	729	20.2	10.7–29.7	98.3	< 0.001
Studies of percutaneous coronary intervention^$^	13	16,873	2357	401	14.1	10.5–17.7	80.3	< 0.001
Studies by countries								
China	2	4324	265	11	3.9	1.6–6.3	0	0.416
Japan	2	1332	199	42	19.9	12.3–27.5	33.4	0.221
Germany	1	458	125	15	12.0	6.3–17.7	–	–
India	1	806	55	5	9.1	1.5–16.7	–	–
Italy	1	208	40	12	30.0	15.8–44.2	–	–
Korea	1	510	74	11	14.9	6.8–23.0	–	–
Turkey	3	3259	430	70	14.6	5.9–23.4	86.4	0.001
United States	6	17,163	1668	7296	35.1	8.0–62.1	99.3	< 0.001
Brazil	1	201	48	4	8.3	0.5–16.2	–	–
Studies by continent								
Asia	10	13,268	1268	173	12.5	7.9–17.2	86.4	< 0.001
Europe	2	666	165	598	19.8	2.3–37.3	81.2	0.021
North America	6	17,163	1668	7296	35.1	8.0–62.1	99.3	< 0.001
South America	1	201	48	4	8.3	0.5–16.2	–	–
Studies by latitude								
North	18	31,097	3101	725	20.9	11.0–30.8	98.4	< 0.001
South	1	201	48	4	8.3	0.5–16.2	–	–
Country income classification^#^								
Lower middle income	1	806	55	5	9.1	1.5–16.7	–	–
Upper middle income	6	7784	743	114	10.0	4.4–15.7	87.2	< 0.001
High income	22	22,708	2351	639	26.4	12.0–40.8	98.6	< 0.001
Country total health expenditure (% of GDP)^&^								
< 5	2	3843	300	39	12.5	8.3–16.7	13.1	0.283
5–10	8	8502	857	108	12.1	6.7–17.5	86.4	< 0.001
> 10	9	18,953	1992	582	28.7	10.1–47.4	98.9	< 0.001
Duration of follow-up								
In-hospital	10	23,301	2118	160	9.4	5.8–13.1	90.4	< 0.001
< 1 year	12	27,144	2348	216	11.6	7.6–15.5	92.4	< 0.001
≥ 1 year	7	18,466	1931	574	29.4	8.6–50.2	99.2	< 0.001

CA-AKI: increase in serum creatinine ≥ 0.5 mg/dl or ≥ 25% from baseline within 72 h after exposure to contrast

^$^Percutaneous coronary intervention: performed from the transfemoral approach according to standard clinical practice

^#^According to the World Bank’s classification of income of countries

^&^According to the World Health Organization

The pooled CA-AKI associated mortality of other two definitions were 20.6% (95% CI 9.1–32.1%) and 27.5% (95% CI 7.8–47.2%) (Supplementary Table 3).

#### Pooled incidence and mortality associated with CA-AKI and variability around the World

**Fig. 3:**
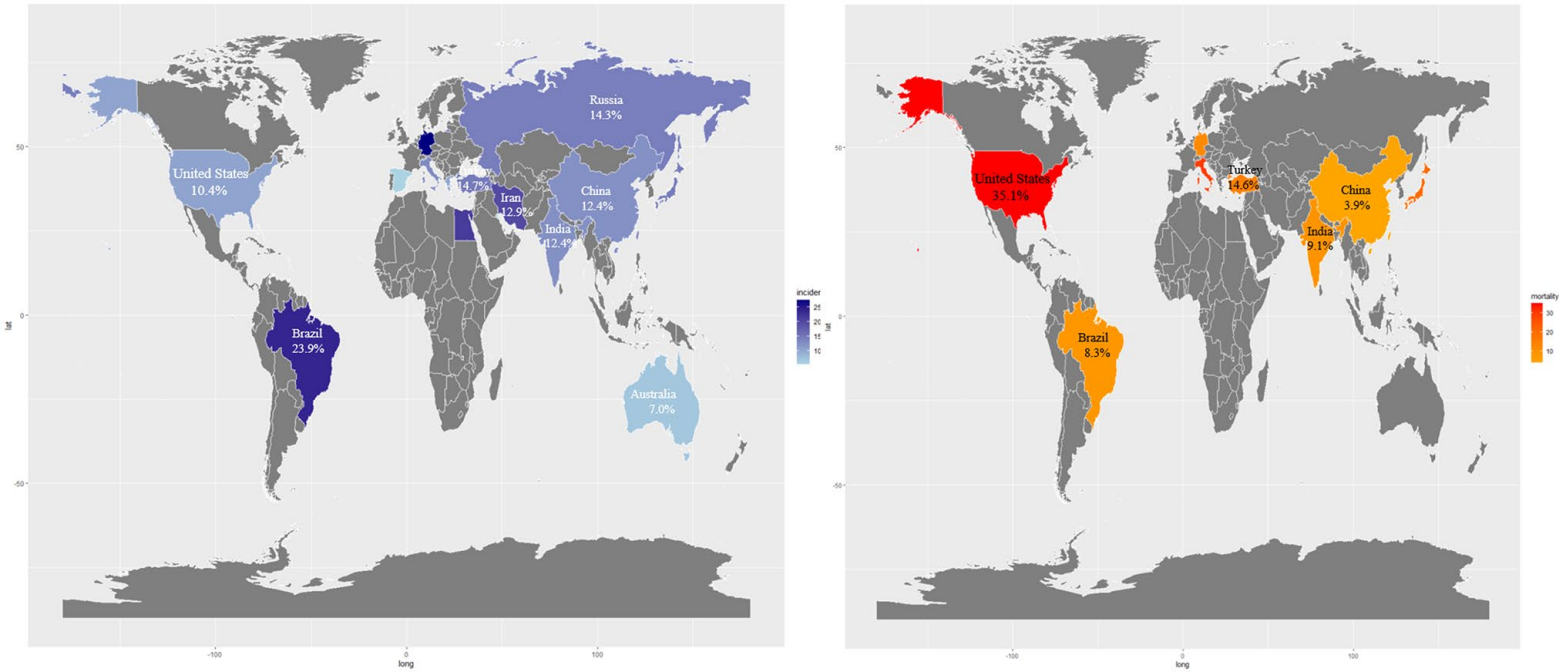
Funnel plot of standard error. CA-AKI: increase in serum creatinine ≥ 0.5 mg/dl or ≥ 25% from baseline within 72 h after exposure to contrast

The incidence rate in Turkey and the United States was 14.7%, in China was 12.4%, in Japan was 14.5% and in Italy was 12.9%. The pooled incidence rate in Asia was higher than in Europe and North America (13.2% versus 12.7% and 10.4%). According to country income classification, the incidence of CA-AKI in high income countries was slightly lower than that of upper middle income countries (11.4% versus 14.1%). And the pooled incidence of CA-AKI in countries with spent over 10% GDP on total health expenditure was less than the countries with 5–10% GDP on total health expenditure (12.2% versus 13.1%). Mortality associated with CA-AKI in Turkey and the United States were 14.6% and 35.1%, China was 3.9%, Japan was 19.9%. Compared with upper middle income countries, the high income countries has higher mortality associated with CA-AKI (26.4% versus 10.0%). The pooled CA-AKI associated mortality in countries with spent over 10% GDP on total health expenditure was higher than the countries with 5–10% GDP on total health expenditure (28.7% versus 12.1%) (Tables 1, 3 and Fig. 3).

## Discussion

Our meta-analysis is the first article with the aim to assess the global incidence of CA-AKI. Since 1946 (the earliest period of Medline retrieval), we have identified a total of 18,868 large cohort studies. The pooled incidence and mortality of CA-AKI after coronary angiography were 12.8% and 20.2%, respectively. Our results showed that the incidence was not significantly related to national economic conditions and the percentage of gross domestic product spent on total health expenditure. And, as time goes on, the incidence of CA-AKI has not changed.

We have pooled the incidence of CA-AKI through 99 studies and found that this is similar to the incidence reported in previous studies [27, 28]. And we also compared different definitions and found that the incidence of other definitions of CA-AKI was both higher than that defined an increase in serum creatinine ≥ 0.5 mg/dl or ≥ 25% from baseline within 72 h after exposure to contrast. Both Chen and Centola’s studies have confirmed that the incidence of CA-AKI is affected by the presence of different definitions [29, 30]. In addition, we also analyzed the incidence of patients with different complications. We found that the incidence of patients with ST-elevation myocardial infarction and chronic kidney disease were higher than the pooled incidence (12.8%), because previous studies have confirmed that ST-elevation myocardial infarction and chronic kidney disease are risk factors of CA-AKI [31, 32]. However, the incidence of CA-AKI in diabetic patients was lower than pooled incidence, which may be related to the small sample size.

We observed that the pooled incidence of CA-AKI in most countries was close to the incidence estimated by us. However, the incidence data reported in Germany, Brazil and Iran were significantly higher, which may be associated with the number of studies and patient characteristics. The patients included in the studies from Germany were elderly, and this was a risk factor for CA-AKI [33, 34]. The increased incidence in Brazil and Iran may be related to the small number of patients included. Because only two studies from Africa and South America were included, it is not possible to draw conclusions from these settings. The incidence rate was 13.2% in Asia, followed by Europe (12.7%) and North America (10.4%, 15 studies were from the United States). The incidence in high income countries was lower than upper middle income countries. Countries that spend more than 10% of GDP on health also have lower rates than countries that spend more than 5–10% of GDP. This may be related to the scale of national economic development and the performance  of the medical and health security system. Moreover, a previous study suggested that AKI had a high incidence in intensive care unit in high income countries, making it difficult to prevent. Conversely, AKI in middle income countries often appears in rural health centers and hospitals as well as in large urban hospitals, and it is possible that it can be prevented through public health initiatives [17]. This may be one of the reasons for the difference. However, the opposite was true for CA-AKI associated mortality, which was higher in high income countries and in countries with high health expenditures as a percentage of GDP. The reason may be that the high mortality rate was at least partly due to other concomitant chronic diseases, which are more frequent in high income countries. Previous studies have confirmed that, in high income countries, patients with AKI were more likely to have risk factors that affect prognosis such as old age, heart failure, diabetes, and hypoproteinemia [35–39].

The incidence of CA-AKI is unclear, which may leads clinicians to underestimate CA-AKI. Our meta-analysis may therefore be of great significance to the future development and formulation of CA-AKI-related public health policies for the scientific community, government, and medical staff. Through meta-regression analysis, we can observe that the incidence and mortality of CA-AKI have not significantly changed since 2002. In addition, we also found that the incidence of CA-AKI from 2014 to 2019 did not change compared with the incidence from 2002 to 2013. Compared with the guidelines on myocardial revascularization of the European Society of Cardiology in 2010, the 2014 guidelines on myocardial revascularization of the European Society of Cardiology recommended that patients who undergo coronary angiography should be assessed for risk of CA-AKI [40, 41]. Moreover, the 2014 guidelines canceled the recommendation of ß-blockers, angiotensin-converting enzyme inhibitors or statins and reduced the level of evidence for *N*-acetylcysteine for chronic kidney disease patients. This may mean that the relevant prevention and treatment strategies of CA-AKI have not achieved actual benefits.

Our meta-analysis had several limitations. Firstly, the studies included in our analysis were targeted to the identification of risk factors of CA-AKI. However, our meta-study included as many as 134 articles, and the sample size was large. Secondly, we had included few studies from Africa and South America, which may lead to the lack of representativeness of results. Studies from the southern hemisphere were also very rare, and more studies are needed in these settings in the future. Thirdly, the various definitions of CA-AKI may have added to the heterogeneity. However, we analyzed the incidence of common definitions of CA-AKI, which makes our results equally representative. Our research compares morbidity and mortality in various countries, but international comparisons may vary depending upon geentic, environmental and social elements.

## Conclusion

According to our metaanalysis, CA-AKI is a common complication in various regions of the world, and incidence and mortality are still high and did not decrease with time. This means that health care managers and and clinicians should pay more attention to CA-AKI to reduce its incidence and improve its prognosis.

## Supplementary Information

Below is the link to the electronic supplementary material.

Supplementary file1 (DOCX 265 kb)

## Data Availability

Not applicable.
