# Correction to: The global incidence and mortality of contrast-associated acute kidney injury following coronary angiography: a meta-analysis of 1.2 million patients

**DOI:** 10.1007/s40620-021-01090-2

**Published:** 2021-06-17

**Authors:** Zhubin Lun, Liwei Liu, Guanzhong Chen, Ming Ying, Jin Liu, Bo Wang, Jingjing Liang, Yongquan Yang, Shiqun Chen, Yibo He, Edmund Y. M. Chung, Jiyan Chen, Jianfeng Ye, Yong Liu

**Affiliations:** 1 Department of Cardiology, Guangdong Provincial Key Laboratory of Coronary Heart Disease Prevention, Guangdong Cardiovascular Institute, Guangdong Provincial People’s Hospital, Guangdong Academy of Medical Sciences, Guangzhou, 510080 China; 2 Department of Cardiology, Dongguan TCM Hospital, Dongguan, 523000 Guangdong China; 3 The First School of Clinical Medicine, Guangdong Medical University, Zhanjiang, 523808 China; 4 Department of Cardiology, Shunde Hospital of Southern Medical University, Shunde, China; 5 Prince of Wales Hospital, Sydney, Australia; 6 Northern Sydney Clinical School, The University of Sydney, Sydney, Australia

## Correction to: Journal of Nephrology 10.1007/s40620-021-01021-1

In the published version of the article the affiliations 1 and 2 has been swapped. The correction affiliations are provided below.

1 Department of Cardiology, Guangdong Provincial Key Laboratory of Coronary Heart Disease Prevention, Guangdong Cardiovascular Institute, Guangdong Provincial People’s Hospital, Guangdong Academy of Medical Sciences, Guangzhou 510,080, China.

2. Department of Cardiology, Dongguan TCM Hospital, Dongguan 523,000, Guangdong, China" to second institution.

The original article has been corrected.

