# Supplementary material for: The global incidence and mortality of contrast-associated acute kidney injury following coronary angiography: a meta-analysis of 1.2 million patients
Source: J Nephrol. 2021 Jun 2;34(5):1479–89. doi: 10.1007/s40620-021-01021-1 (PMC8494686; doi:10.1007/s40620-021-01021-1)
Supplement: Supplementary file 1 — Supplementary file1 (DOCX 265 kb) [file 40620_2021_1021_MOESM1_ESM.docx]

**Search strategy**

A systematic computer-aided search of related studies will be conducted in the following databases:

1. Ovid MEDLINE (1946 to June 30, 2019, including epub ahead of print, in process and other non-indexed citations and daily)

2. Ovid Embase (1947 to June 2019)

3. Cochrane Database of Systematic Reviews （published on or before 30th June 2019）

1, Search strategy for MEDLINE

| **Ovid MEDLINE(R) <1946 to June 30 2019>** | | | |
| --- | --- | --- | --- |
| # | **Retrieval content** | **Results** | **Comments** |
| 1 | exp Contrast Media/ | 115065 |  |
| 2 | (contrast media or contrast medium or contrast material$ or contrast agent$ or contrast dye or radiographic contrast).ti,ab. | 54801 |  |
| 3 | (radiocontrast media or radiocontrast medium or radiocontrast agent$).ti,ab. | 581 |  |
| 4 | (percutaneous coronary intervention or PCI or coronary angiograph or angiography or catheter-proven or Angioplasty).ti,ab. | 201133 |  |
| 5 | 1 or 2 or 3 or 4 | 324018 |  |
| 6 | (nephritis or nephropath$ or nephrotoxic$).ti,ab. | 93809 |  |
| 7 | ((impair$ or damag$ or reduc$ or injur$ dysfunction$ or failure) adj2 (renal or kidney)).ti,ab. | 131263 |  |
| 8 | exp Kidney Diseases/ | 493327 |  |
| 9 | exp nephritis/ or diabetic nephropathies/ | 94877 |  |
| 10 | exp renal insufficiency/ | 162870 |  |
| 11 | (acute kidney injury or acute kidney injur* or acute kidney failure* or Acute kidney insufficienc* or contrast induced nephropathy or contrast induced nephropath* or contrast nephropath* or contrast induced acute kidney injur* or AKI or ARF or CI-AKI or acute renal injur* or acute renal failure* or acute renal insufficienc* or creatinine or serum creatinine).ti,ab. | 156293 |  |
| 12 | 6 or 7 or 8 or 9 or 10 or 11 | 651329 |  |
| 13 | Risk Assessment/ | 244445 |  |
| 14 | risk factors/ | 774485 |  |
| 15 | ((risk adj3 model*) or score* or prognostic factor* or predict* or regression* or (logistic adj2 model*) or multivariable logistic regression or multivariable analyses or logistic regression or algorithm* or equation* or (multivariate adj3 analysis)).ti,ab. | 2935283 |  |
| 16 | Forecasting/ | 82331 |  |
| 17 | "Predictive Value of Tests"/ | 192289 |  |
| 18 | multivariate analysis/ | 120489 |  |
| 19 | sn.fs. | 864532 |  |
| 20 | exp mathematical concepts/ | 938358 |  |
| 21 | exp Models, Biological/ | 785414 |  |
| 22 | exp models, statistical/ | 386318 |  |
| 23 | area under curve/ | 37993 |  |
| 24 | 13 or 14 or 15 or 16 or 17 or 18 or 19 or 20 or 21 or 22 or 23 | 5428233 |  |
| 25 | 5 and 12 and 24 | 7291 |  |

**2, Search strategy for Embase**

| **Embase <1974 to 2019 June 30>** | | | |
| --- | --- | --- | --- |
| # | **Retrieval content** | **Results** | **Comments** |
| 1 | 'contrast media'/exp | 135472 |  |
| 2 | ('contrast media':ab,ti OR 'contrast medium':ab,ti OR 'contrast material$':ab,ti OR 'contrast agent$':ab,ti OR 'contrast dye':ab,ti OR 'radiographic contrast':ab,ti) | [57,814](https://www.embase.com/) |  |
| 3 | ('radiocontrast media':ab,ti OR 'radiocontrast medium':ab,ti OR 'radiocontrast agent$':ab,ti) | [695](https://www.embase.com/) |  |
| 4 | ('percutaneous coronary intervention':ab,ti OR 'pci':ab,ti OR 'coronary angiograph':ab,ti OR 'angiography':ab,ti OR 'catheter-proven':ab,ti OR 'angioplasty':ab,ti) | [267,579](https://www.embase.com/) |  |
| 5 | #1 OR #2 OR #3 OR #4 | [442,485](https://www.embase.com/) |  |
| 6 | ('nephritis':ab,ti OR 'nephropath$':ab,ti OR 'nephrotoxic$':ab,ti) | [88,815](https://www.embase.com/) |  |
| 7 | ('impair$' OR 'damag$' OR 'reduc$' OR 'injur$ dysfunction$' OR 'failure') AND ('renal':ab,ti OR 'kidney':ab,ti) | [341,487](https://www.embase.com/) |  |
| 8 | 'kidney diseases'/exp | [783,031](https://www.embase.com/) |  |
| 9 | ('nephritis'/exp OR 'diabetic nephropathies'/exp) | [116,007](https://www.embase.com/) |  |
| 10 | 'renal insufficiency'/exp | [327,715](https://www.embase.com/) |  |
| 11 | ('acute kidney injury':ab,ti OR 'acute kidney injur*':ab,ti OR 'acute kidney failure*':ab,ti OR 'acute kidney insufficienc*':ab,ti OR 'contrast induced nephropathy':ab,ti OR 'contrast induced nephropath*':ab,ti OR 'contrast nephropath*':ab,ti OR 'contrast induced acute kidney injur*':ab,ti OR 'aki':ab,ti OR 'arf':ab,ti OR 'cin':ab,ti OR 'acute renal injur*':ab,ti OR 'acute renal failure*':ab,ti OR 'acute renal insufficienc*':ab,ti OR 'creatinine':ab,ti OR 'serum creatinine':ab,ti) | [215,577](https://www.embase.com/) |  |
| 12 | #6 OR #7 OR #8 OR #9 OR #10 OR #11 | [946,919](https://www.embase.com/) |  |
| 13 | 'risk assessment' | [473,172](https://www.embase.com/) |  |
| 14 | 'risk factor' | [853,042](https://www.embase.com/) |  |
| 15 | ('risk model*':ab,ti OR 'score*':ab,ti OR 'prognostic factor*':ab,ti OR 'predict*':ab,ti OR 'regression*':ab,ti OR 'logistic model':ab,ti OR 'multivariable logistic regression':ab,ti OR 'multivariable analyses':ab,ti OR 'logistic regression':ab,ti OR 'algorithm*':ab,ti OR 'equation*':ab,ti OR 'multivariate analysis':ab,ti) | [3,327,622](https://www.embase.com/) |  |
| 16 | forecasting | [11,584](https://www.embase.com/) |  |
| 17 | 'predictive value of tests' | [606](https://www.embase.com/) |  |
| 18 | 'multivariate analysis' | [238,859](https://www.embase.com/) |  |
| 19 | 'mathematical concepts' | [3,339,225](https://www.embase.com/) |  |
| 20 | 'models, biological' | [1,293,818](https://www.embase.com/) |  |
| 21 | 'models, statistical' | [62,844](https://www.embase.com/) |  |
| 22 | 'area under curve' | [124,515](https://www.embase.com/) |  |
| 23 | #13 OR #14 OR #15 OR #16 OR #17 OR #18 OR #19 OR #20 OR #21 OR #22 | [7,090,960](https://www.embase.com/) |  |
| 24 | #5 AND #12 AND #23 | [16,966](https://www.embase.com/) |  |

**3, Cochrane Central**

**Cochrane review search < 2019 June 30>**

| **Retrieve content** | | **Result** | **Comments** |
| --- | --- | --- | --- |
| 1 | (Contrast Media):ti,ab,kw | 78 |  |
| 2 | (contrast media OR contrast medium OR contrast material$ OR contrast agent$ OR contrast dye OR radiographic contrast):ti,ab,kw | 122 |  |
| 3 | (radiocontrast media or radiocontrast medium or radiocontrast agent$):ti,ab,kw | 0 |  |
| 4 | (percutaneous coronary intervention OR PCI OR coronary angiograph OR angiography OR catheter-proven or Angioplasty):ti,ab,kw | 88 |  |
| 5 | 1 or 2 or 3 or 4 | 179 |  |
| 6 | (nephritis OR nephropath$ OR nephrotoxic$):ti,ab,kw | 24 |  |
| 7 | (impair$ OR damag$ OR reduc$ OR injur$ OR dysfunction$ OR failure):ti,ab,kw AND (renal OR kidney):ti,ab,kw | 419 |  |
| 8 | (Kidney Diseases):ti,ab,kw | 271 |  |
| 9 | (nephritis):ti,ab,kw OR (diabetic nephropathies):ti,ab,kw | 20 |  |
| 10 | (renal insufficiency):ti,ab,kw | 132 |  |
| 11 | (acute kidney injury OR acute kidney injur* OR acute kidney failure* OR Acute kidney insufficienc* OR contrast induced nephropathy OR contrast induced nephropath* OR contrast nephropath* OR contrast induced acute kidney injur* OR AKI OR ARF OR CI-AKI OR acute renal injur* OR acute renal failure* OR acute renal insufficienc* OR creatinine OR serum creatinine):ti,ab,kw | 151 |  |
| 12 | 6 or 7 or 8 or 9 or 10 or 11 | 330 |  |
| 13 | (Risk Assessment):ti,ab,kw | 5657 |  |
| 14 | (risk factors):ti,ab,kw | 1349 |  |
| 15 | (risk AND model*):ti,ab,kw OR (score* OR prognostic factor* OR predict* OR regression*):ti,ab,kw OR (logistic AND model*):ti,ab,kw OR (multivariable logistic regression OR multivariable analyses OR logistic regression OR algorithm* OR equation*):ti,ab,kw OR (multivariate AND analysis):ti,ab,kw | 2987 |  |
| 16 | (Forecasting):ti,ab,kw | 2 |  |
| 17 | (Predictive Value of Tests) | 2223 |  |
| 18 | (multivariate analysis) | 396 |  |
| 19 | sn.fs. | ?? |  |
| 20 | (mathematical concepts):ti,ab,kw | 34 |  |
| 21 | (Models AND Biological):ti,ab,kw | 34 |  |
| 22 | (models, statistical):ti,ab,kw | 783 |  |
| 23 | (area under curve) | 631 |  |
| 24 | 13 or 14 or 15 or 16 or 17 or 18 or 19 or 20 or 21 or 22 or 23 | 4548 |  |
| 25 | 5 and 12 and 24 | 6 |  |

**Supplementary Table 1. Characteristics of the included studies.**

| **Cohort studies** | **Country/ Study period** | **Continents** | **Procedure** | **Cohort size** | **Number of CI-AKI in cohort** | **CI-AKI definition** |
| --- | --- | --- | --- | --- | --- | --- |
|  |  |  |  |  |  |  |
|  |  |  |  |  |  |  |
|  |  |  |  |  |  |  |
| Abdurrezzak Börekçi, 2014[1] | Turkey, 2013-2014 | Asia | Percutenous coronary intervention | 289 | 69 | Contrast-induced nephropathy is Defined as an increase of 25% or more in serum creatinine concentrations from baseline within 72 hours following primary angioplasty |
| Ahmet Kaya, 2018[2] | Turkey, 2015-2018 | Asia | Emergent PCI | 963 | 128 | CI-AKI was Defined as pre viously described and distinguished as grade 0 (serum creatinine increase <25% above baseline and <0.5 mg/dL above baseline), grade 1 (serum creatinine increase ≥25% above baseline and <0.5 mg/dL above baseline), or grade 2 (serum creatinine increase ≥0.5 mg/dL above baseline) |
| Ali Amiri, 2018[3] | Iran, 2007-2010 | Asia | Elective CAG or PCI | 255 | 69 | Defined as an absolute or relative increase in SCr to ≥0.5 mg/dL (44 μmol/L) or ≥25% above baseline within 48 h after angiography, respectively |
| Alparslan Kurtul, 2017[4] | Turkey, 2013-2016 | Asia | Emergent PCI | 1408 | 159 | Defined as a rise in serum creatinine >0.5 mg/dL or >25% increase in baseline within 72 hours after PCI |
| Amal Abdel Ghani, 2009[5] | Kuwait, 2008-2009 | Asia | Percutenous coronary intervention | 247 | 13 | Defined as an increase in serum creatinine concentration of ≥ 44.2 µmol/L within 48 hours after the procedure. |
| C. Tang, 2019[6] | China, 2017-2018 | Asia | Emergent PCI | 240 | 29 | CI-AKI is Defined as an increase in serum creatinine of more than 25% or 44.2 mmol/L−1 48 to 72 hours afer contrast medium adminis tration without evidence of other causes |
| Charanjit S, 2002[7] | USA, 2009-2010 | North America | Coronary angiography or percutaneous coronary intervention | 7586 | 254 | Defined as increase in serum creatinine (Cr) concentration of ≥0.5 mg/dL from preprocedure values |
| Chunrui Wang, 2017[8] | China, 2010-2011 | Asia | Elective CAG or PCI | 912 | 50 | Defined as a 44.2 lmol/L rise in serum creatinine or a 25% increase, assessed within 48 h after administration of contrast |
| D. M. Leistner, 2018[9] | Germany, 2009-2014 | Europe | Percutenous coronary intervention | 458 | 125 | AKI was defined as an absolute increase of serum creatinine of ≥0.5 mg/dL or a relative increase of ≥25% from baseline values within the first 72 h following administration of contrast medium |
| David R. McIlroy, 2012[10] | USA, 2008-2009 | North America | Coronary angiogrphy | 644 | 141 | Defined as an increase in serum creatinine by 0.3 mg/dL or 50% above baseline within the first 2 postoperative days or the commencement of RRT within the same time period |
| Denis F. Souza, 2015[11] | Brazil, 2007-2010 | Others | Elective CAG | 125 | 22 | Defined as an increase in sCr concentration of 0.3 mg/dl over baseline 48 h after the infusion of contrast media in patients who did not develop AKI to advanced stages. |
| Dimitrios Tziakas, 2011[12] | Greece, 2012-2015 | Europe | Percutenous coronary intervention | 488 | 50 | Defined as increase ≥25% and/or ≥0.5 mg/dl in serum creatinine at 48 h after PCI vs baseline |
| E. Izkhakov, 2019[13] | Israel, 2014-2017 | Asia | Emergent PCI | 723 | 64 | AKI was determined using the Kidney Disease: Improving Global Outcomes criteria and Defined as a sCr rise >0.3 mg/dL within 48 h of contrast exposure compared with admission sCr |
| Eric Chong, 2010[14] | Singapore, 1996-2007 | Asia | Percutenous coronary intervention | 3,036 | 222 | CI-AKI was defined as >25% or >0.5 mg/dL increase from baseline Cr within 48 hours after PCI |
| Eugenia Nikolsky, 2005[15] | USA, 6-year period | North America | Percutenous coronary intervention | 6,773 | 942 | Defined as increase of ≥25% or ≥0.5 mg/dL |
| Eyup Avci, 2017[16] | Turkey, 2004-2005 | Asia | Coronary angiogrphy | 214 | 43 | Defined as impairment of renal function and characterized by a 25% or greater increase in serum creatinine from baseline to within 48 h of intravenous contrast media administration |
| Fei Gao, 2011[17] | China, 2005-2007 | Asia | Percutenous coronary intervention | 4,522 | 726 | Defined as an absolute serum creatinine increase ≥44 μmol/l or a relative increase in serum creatinine ≥25% occurring within 48–72 h after the coronary procedure |
| Fei He, 2012[18] | China, 2010-2011 | Asia | Percutenous coronary intervention | 325 | 51 | Defined as AKIN(AKI network) criteria |
| Feldkamp T, 2017[19] | Germany, 2009-2010 | Europe | Percutenous coronary intervention | 2,937 | 400 | Defined as an increase of the SCr level by 0.3 mg/dl within 48 h or an increase to at least 150% of the value prior to the baseline |
| Felipe Hernández, 2009[20] | Spain, 2005-2007 | Europe | Coronary angiography or percutaneous coronary intervention | 250 | 14 | Defined as an absolute increase in SCr from baseline of >0.5 mg/dL or a relative increase of >25% at 72 hours following exposure to CM |
| Feng Hua Ding, 2013[21] | China, 2011-2013 | Asia | Coronary angiography or percutaneous coronary intervention | 1,030 | 114 | Defined as ≥25% increase in serum creatinine within 72 h after the procedure |
| Florian Lüders, 2015[22] | Germany, 2006-2009 | Europe | Elective PCI | 373 | 79 | Defined and classified according to the guidelines of the Acute Kidney Injury Network (AKIN) |
| G. Cicek, 2017[23] | Turkey, UK | Asia | Emergent PCI | 2,972 | 693 | Defined as 25% or higher elevation in the basal creatinine value or 0.5 mg/dL or higher elevation in the creatinine concentration |
| George Dangas, 2005[24] | USA, 2004-2005 | North America | Percutenous coronary intervention | 7,230 | 1,069 | Defined as an increase of ＞25% and/or ＞0.5 mg/dl in preprocedure serum creatinine at 48 hours after the procedure |
| Giancarlo Marenzi, 2004[25] | Italy, 2001-2003 | Europe | Emergent PCI | 208 | 40 | Defined as an absolute increase in Cr > 0.5 mg/dl after PCI |
| Giuseppe Ando, 2013[26] | Italy, 2008-2011 | Europe | Percutenous coronary intervention | 481 | 25 | Defined as an absolute increase in serum creatinine ≥0.5 mg/dL or an increase ≥25% from baseline within 72 hr |
| Guoqiang Gu, 2017[27] | China, 2014-2016 | Asia | Percutenous coronary intervention | 431 | 25 | Defined as either a 25% relative increase or an absolute increase of 0.5 mg/dL (44.2 µmol/L) in the serum creatinine (Scr) level at 48–72 h after administration of iodinated contrast media |
| Gustavo N. Araujo, 2017[28] | Brazil, 2011-2015 | Others | Emergent PCI | 347 | 46 | Defined as a raise of 0.3 mg/dL or 50% in post procedure (24–72 h) creatinine compared to baseline, proposed by the Acute Kidney Injury Network (AKIN) as a standardized definition of acute kidney injury |
| Hakan Ucar, 2013[29] | Turkey, 2013-2013 | Asia | Percutenous coronary intervention | 440 | 78 | Defined as an increase of 25% in serum creatinine concentrations from baseline within 72 hours following primary angioplasty. |
| Han B, 2018[30] | China, 2015-2016 | Asia | Emergent PCI | 379 | 88 | Defined as an increase of≥44.2 μmol/L or≥25% in serum creatinine level during the frst 72 h following angiography |
| Hiroaki Watabe, 2014[31] | Japan, 2007-2010 | Asia | Emergent PCI | 1,278 | 164 | Defined as impairment in renal function resulting in a ≥0.5 mg/dL and/or ≥25% absolute increase in serum creatinine from baseline |
| Hossein Nough, 2013[32] | Iran, 2011-2013 | Asia | Coronary angiography or percutaneous coronary intervention | 250 | 32 | Defined by an increase in creatinine of >0.5 mg/dl or 25% of the initial value |
| Hyuck-Jun Yoon, 2013[33] | Korea, 2009-2010 | Asia | Coronary angiography or percutaneous coronary intervention | 723 | 29 | Defined as a 25% elevation or an absolute increase of >0.5mg/dL (>44 umol/L) in the sCr level compared to the baseline within 48 to 72 hours after PCI |
| Irfan Sahin, 2013[34] | Japan, 2009-2011 | Asia | Elective CAG | 403 | 74 | Defined as the elevation of serum creatinine levels by > 0.5 mg/dL or > 25% occurring within 48-72 hours after the intravascular administration of CM, without another cause |
| J. A. Dodson, 2019[35] | USA, 2013-2016 | North America | Emergent PCI | 2,212 | 421 | Kidney Disease Improving Global Outcomes (KDIGO) criteria (serum Cr increase ≥0.3 mg/dL from baseline or ≥1.5 times baseline) |
| Jae Hee Kim, 2014[36] | Korea, 2005-2011 | Asia | Emergent PCI | 971 | 93 | Defined using absolute change in serum creatinine (SCr; SCr <24 hours after primary PCI minus admission SCr) as follows: no early AKI (SCr change <0.3 mg/dl), mild early AKI (SCr change 0.3 to <0.5 mg/dl), moderate early AKI (SCr change 0.5 to <1.0 mg/dl), and severe early AKI (SCr change ‡1.0 mg/dl) |
| Jae Yeong Cho, 2010[37] | Korea, 2004-2005 | Asia | Coronary angiography or percutaneous coronary intervention | 510 | 74 | Defined as an increase of ≥25% or ≥0.5 mg/dL in pre-procedure serum Cr after procedure. |
| Javier A. Neyra, 2013[38] | USA, 2008-2009 | North America | Coronary angiography or percutaneous coronary intervention | 1,160 | 216 | Defined as SCr either ≥ 25% or ≥ 0.5 mg/dL from baseline within 72 h after contrast exposure. |
| Jayakumar Sreenivasan, 2018[39] | USA, 2012-2016 | North America | Percutenous coronary intervention | 2,055 | 293 | Defined CI-AKI as ≥0.3 mg/dL rise in creatinine from baseline within 48 hours of exposure to contrast based on this most recent and widely accepted KDIGO consensus definition of AKI |
| Jin Wi, 2012[40] | Korea, 2005-2009 | Asia | Percutenous coronary intervention | 1,041 | 148 | Defined as >25% or >0.5 mg/dL increase in serum creatinine level within 48 hours after administration of contrast medium when no other major kidney insult was identified. |
| Julio G. Peguero, 2013[41] | USA, 2006-2009 | North America | Percutenous coronary intervention | 199 | 43 | Defined as either a 25% or a 0.5 mg/dL, or greater, increase in serum creatinine during the first 48 to 72 hours after contrast exposure |
| Kang Hyu Lee, 2010[42] | Korea, 2005-2006 | Asia | Percutenous coronary intervention | 537 | 51 | Defined as a rise in serum creatinine of ≥25% or ≥0.5 mg/dL above the baseline value within 48 hours after contrast administration |
| Kato K, 2008[43] | Japan, 2005-2005 | Asia | Elective CAG or PCI | 87 | 18 | Defined as an increase of more than 25% from the baseline value of serum Cr, or an absolute increase of at least 0.5 mg/dl (44.2μmol/L) within 48 h after the administration of contrast medium. |
| Kosei Tanaga, 2012[44] | Japan, 2005-2006 | Asia | Coronary angiography or percutaneous coronary intervention | 300 | 18 | Defined as a serum creatinine increase of 25 % and/or 0.5 mg/dL over 48 h after exposure to the contrast medium |
| L. S. Nguyen, 2018[45] | France, UK | Europe | Emergent PCI | 701 | 84 | the presence of CI-AKI 48 hours after admission using the Acute Kidney Injury Network (AKIN) definition (a +0.3 mg/dL [26.5mol/L] or + 50% relative increase in serum creatinine) |
| Ling Ji, 2015[46] | China, 2011-2013 | Asia | Percutenous coronary intervention | 565 | 68 | Defined as an increase of >25% or >0.5mg/dl (44.2μmol/L) in baseline serum creatinine level within 72 hours after the use of contrast agents, without other factors lead to renal impairment |
| Ling Sun, 2018[47] | China, 2011-2013 | Asia | Coronary angiography or percutaneous coronary intervention | 751 | 106 | Defined as "an absolute increase of serum creatinine of more than or equal to 0.3mg/dL or increase to more than or equal to 150% from baseline within any 48 hour during hospital days" |
| Lucia Barbieri, 2016[48] | Italy, 2007-2013 | Europe | Coronary angiogrphy | 2,851 | 359 | Defined as an absolute 0.5mg/dL or a relative 25% increase in creatinine level 24 to 48 hours after the procedure. |
| Masaomi Gohbara, 2017[49] | Japan, 2010-2016 | Asia | Emergent PCI | 273 | 35 | Defined as an increase of 0.5 mg/dL in serum creatinine or a 25% increase from baseline between 48 and 72 hours after contrast medium exposure |
| Mendi MA, 2016[50] | Turkey, 2010-2012 | Asia | Percutenous coronary intervention | 450 | 73 | Defined a increase of ≥25% or absolute increase of ≥0.5 mg/dL in creatinine concentrations within 72 hours after PCI |
| Min Young Rim, 2012[51] | Korea, 2003-2010 | Asia | Coronary angiography or percutaneous coronary intervention | 5,299 | 233 | Defined by AKI Network (AKIN) criteria: an absolute increase in serum creatinine levels ＞0.3 mg/dL or a relative increase 50% from baseline values within 48 hours after exposure to the contrast medium. |
| Mitsuru Abe, 2009[52] | Japan, 2003-2004 | Asia | Coronary angiography or percutaneous coronary intervention | 1,157 | 161 | Defined as concentration ≥0.5 mg/dl or 25% within 5 days after |
| Murat Saritemur, 2014[53] | Japan, 2005-2006 | Asia | Emergent PCI | 744 | 93 | Defined as an acute deterioration of renal function in which an increase in serum Cr level of 25% or 0.5 mg/dL above the baseline value occurs within 48 hours after the administration of a contrast medium without an alternative etiology |
| Naikuan Fu, 2012[54] | China, 2007-2010 | Asia | Coronary angiography or percutaneous coronary intervention | 668 | 105 | Defined as a relative elevation ≥25% in SCr or an absolute increase of 0.5 mg/dL (44.2 mmol/L), or a combination of the both, at 48 to 72 hours after exposure to a contrast agent compared to the baseline SCr values, without alternative explanations for renal impairment. |
| Omer Celik, 2014[55] | Turkey, UK | Asia | Emergent PCI | 597 | 78 | Defined as an absolute 0.3 mg/dL increase in serum creatinine compared with baseline levels within 48 hours after the procedure was considered as CI-AKI |
| Omer Toprak, 2006[56] | Turkey, 2004-2005 | Asia | Elective CAG | 266 | 23 | Defined as an increase of 25% in creatinine over baseline within 48 hr of angiography |
| Omer Toprak, 2007[57] | Turkey, 2011-2013 | Asia | Elective CAG | 421 | 52 | Defined as an increase of 25% in creatinine over baseline within 48 h of angiography |
| Nzgür Günebakmaz, 2013[58] | Turkey, 2005-2006 | Asia | Coronary angiogrphy | 90 | 10 | Defined as an increase ≥0.5 mg/dl and/or ≥25% in SCr concentration at day 2 of the contrast exposure |
| Pamela S. Reed, 2007[59] | USA, 2004-2005 | North America | Coronary angiography or percutaneous coronary intervention | 169 | 28 | Defined as an increase of 0.5 mg/dl in serum creatine from baseline |
| Paulo Roberto Santos, 2015[60] | Brazil, 2013-2014 | Others | Emergent PCI | 201 | 48 | Defined as an absolute increase of creatinine of at least 0.5 mg/ dL and/or a relative increase of creatinine of 25% in relation to baseline in a period between 48 and 72 hours after contrast administration. |
| Praveen Kandula, 2010[61] | USA, 2009-2014 | North America | Elective PCI | 353 | 75 | Defined as an increase of >25% or >44 mmol/L in baseline serum creatinine at 48–72 h following PCI |
| Probal Roy, 2008[62] | USA, 2004-2006 | North America | Percutenous coronary intervention | 570 | 70 | Defined as a >25% increase in baseline creatinine |
| Qin YH, 2018[63] | China, 2011-2016 | Asia | Coronary angiogrphy | 258 | 45 | Defined as diagnosed based on an increase in serum creatinine by >25% or by 44.2 μmol/l within 48 72 h of coronary angiography after excluding other factors |
| Roxana Mehran, 2004[64] | USA, 2011-2013 | North America | Percutenous coronary intervention | 5,571 | 729 | Defined as increase of≥25% or ≥0.5 mg/dl in pre-PCI serum creatinine at 48 h after PCI |
| Rudolf Jarai, 2012[65] | USA, / | North America | Percutenous coronary intervention | 979 | 131 | Defined as a relative increase of ≥25% or absolute increase of ≥0.5 mg/dL in creatinine concentrations within 48 hours after index angiography |
| Ruey-Hsing Chou, 2016[66] | Taiwan, China, 2011-2013 | Asia | Elective PCI | 539 | 55 | Defined as the elevation of serum Cr ≥0.5 mg/dL or≥25% in baseline serum Cr |
| Rui Wu, 2018[67] | China, 2015-2016 | Asia | Coronary angiogrphy | 460 | 125 | Defined as any of the following: an increase in SCr by ≥0.3mg/dl (≥26.5μmol/l) within 48 hours; or an increase in SCr to ≥1.5 times baseline, which is known or presumed to have occurred within the prior 7 days; or urine volume <0.5ml/kg/h for 6 hours |
| S. Sigirci, 2019[68] | Turkey, 2015-2017 | Asia | Emergent PCI | 883 | 126 | Defined as an increase in serum creatinine level of 0.5 mg/dL or 25% above baseline within 72 hours after contrast administration |
| Saito Y, 2015[69] | Japan, 2011-2013 | Asia | Percutenous coronary intervention | 906 | 45 | Defined as increase in serum creatinine ≥ 0.5 mg/dl or ≥ 25% from baseline between 48 and 72 h after exposure to contrast. |
| Salvatore Evola, 2012[70] | Italy, 2008-2009 | Europe | Coronary angiography or percutaneous coronary intervention | 591 | 105 | Defined as an absolute increase in serum creatinine of 25% from baseline values occurring within 24-48 h after the coronary procedure |
| Samuel Goussot, 2015[71] | France, 2001-2013 | Europe | Percutenous coronary intervention | 1,243 | 130 | Defined according to current Kidney Disease : Improving Global outcomes (KDIGO) guidelines endorsed by European Renal Best Practice (ERBP) working group by an absolute (> 26.5 μmol/L or 0.3 mg/dL) or a relative (> 1.5) increase in SCL from baseline within 48 H-72H after injection of the CM. |
| Sanjai Pattu Valappil, 2017[72] | India, 2007-2010 | Asia | Elective PCI | 100 | 29 | Defined as ncrease in serum creatinine concentration of 0.5 mg/dL (44 mol/L) or 25% above baseline within 48 h after contrast administration |
| Serdar Farhan, 2015[73] | Austria, 2003-2006 | Europe | Percutenous coronary intervention | 536 | 51 | CI-AKI was diagnosed according to risk, injury, failure, loss of kidney function and end-stage kidney disease/acute kidney injury network (RIFLE/AKIN) criteria |
| Shao-Sung Huang, 2012[74] | Taiwan, China, 2005-2006 | Asia | Percutenous coronary intervention | 544 | 85 | Defined as a rise of SCr of 0.5 mg/dl or a 25% increase from the baseline value within 48 hours after PCI |
| Silvia Esmeralda Pérez-Topete, 2016[75] | Russia, 2004-2005 | Europe | Percutenous coronary intervention | 70 | 10 | Defined as: the impairment of renal function and is measured as either a 25% increase in serum creatinine (SCr) from baseline or 0.5 mg/dL increase in absolute value, within 48-72 hours of intravenous contrast administration |
| Suhas S. Lele, 2013[76] | India, 2011-2013 | Asia | Coronary angiography or percutaneous coronary intervention | 806 | 55 | Defined as an absolute elevation in serum creatinine of ≥0.5 mg/dL from baseline within the first 48 hours after contrast exposure. |
| Taeyoung Kong, 2017[77] | Korea, 2011-2016 | Asia | Emergent PCI | 564 | 58 | Defined as absolute (≥0.5 mg/dL) or relative (>25%) increase in SCr from baseline on ED admission within 48 to 72 hours afterexposure of contrast medium |
| Takeshi Senoo, 2010[78] | Japan, 2012-2015 | Asia | Emergent PCI | 338 | 94 | Defined as an 25% increase in SCr from baseline or an absolute increase of≥0.5 mg/dl that appeared within 2 days after emergency PCI |
| Taner Ulus, 2018[79] | Turkey, 2005-2006 | Asia | Percutenous coronary intervention | 647 | 70 | Defined as the elevation of serum creatinine 0.5 mg/dL or 25% in baseline serum creatinine within 72 hours after PCI. |
| Thomas T. Tsai, 2014[80] | USA, 2004-2005 | North America | Percutenous coronary intervention | 985,737 | 69,658 | Defined as using the AKIN criteria (AKI stage 1, ≥ 0.3 mg/dl absolute or 1.5 to 2.0-fold relative increase in serum creatinine; AKI stage 2, >2- to 3-fold increase in serum creatinine; AKI stage 3, >3-fold increase in serum creatinine or serum creatinine >4.0 mg/dl with an acute increase of >0.5 mg/dl). |
| Toshiki Kuno, 2018[81] | Japan, 2004-2005 | Asia | Percutenous coronary intervention | 8,782 | 838 | Defined as an absolute increase in serum creatinine values of 0.3 mg/dL or a relative increase of 50%, in accordance with the Acute Kidney Injury Network criteria |
| Victor SM, 2014[82] | India, 2008-2009 | Asia | Percutenous coronary intervention | 900 | 87 | Defined as an increase of ≥ 25% and/or ≥ 0.5 mg/dl in serum creatinine at 48 hours after PCI when compared to baseline value. |
| Vojko Kanic, 2018[83] | Slovenia, 2007-2016 | Europe | Emergent PCI | 5,859 | 499 | AKI was determined using the KDIGO criteria, as follows: stage 1 – a rise in serum creatinine by ≥0.3 mg/dL within 48 h, or an increase in serum creatinine of at least 1.5 times the baseline that is known or presumed to have occurred within the prior 7 days; stage 2 – more than a 2- to 2.9-fold increase in serum creatinine; stage 3 – more than a 3-fold increase in serum creatinine from the baseline or an increase in serum creatinine to >4.0 mg/dL |
| Wang Ling, 2008[84] | China, 2007-2010 | Asia | Coronary angiogrphy | 150 | 13 | Defined as diagnosed as an increase in SCr >=44.2 mol/l or 25% from baseline 48–72 h after the procedure |
| Warren K. Laskey, 2007[85] | USA, 2007-2010 | North America | Percutenous coronary intervention | 3,179 | 48 | Defined as An increase in serum creatinine of ＞ 0.5 mg/dl by 24 to 48 h |
| Wuhua Jiang, 2018[86] | China, 2009-2010 | Asia | Coronary angiography or percutaneous coronary intervention | 1,069 | 412 | Defined as: increase in serum creatinine (SCr) by ≥0.3 mg/dL (≥26.5 μmol/L) within 48 h; or increase in SCr to ≥1.5 times baseline that is known or presumed to have occurred within the prior 7 days or urine volume < 0.5 mL/kg per hour for 6 h. |
| Xi-peng Sun, 2017[87] | China, 2005-2006 | Asia | Emergent PCI | 5,719 | 252 | Defined as ≥25% relative increase or ≥0.5 mg/dL absolute increase in serum creatinine level above baseline within 72 hours of contrast exposure, in the absence of an alternative explanation |
| Xun Hu, 2016[88] | China, 2014-2015 | Asia | Elective PCI | 192 | 32 | Defined as an increase in serum creatinine of 0.5 mg/dL within 48-72 hours after the procedure. |
| Y.R. Sedhai, 2017[89] | USA, 2014 | North America | Coronary angiography or percutaneous coronary intervention | 418 | 16 | Defined as an increase in serum creatinine concentration by ≥25% or ≥0.5mg/dl from the pre-procedural value within 72 hours of contrast exposure |
| Yalcin Velibey, 2016[90] | Turkey, 2005-2006 | Asia | Emergent PCI | 2,563 | 164 | Defined as ≥25% relative increase or ≥0.5 mg/dL absolute increase in serum creatinine above baseline within 72 hours after pPCI |
| Yijie Hu, 2013[91] | China, 2010-2012 | Asia | Coronary angiogrphy | 71 | 22 | Defined as an absolute increase of 0.3 mg/dL or a relative increase of 50%in serum creatinine from baseline values |
| You ZB, 2018[92] | China, 2012-2015 | Asia | Elective PCI | 558 | 51 | Defined as an absolute increase in >0.3 mg/dL or >50% from the baseline SCr levels within 48 h after exposure to CM |
| YousUKe Taniguchi, 2012[93] | Japan, 2006-2009 | Asia | Elective CAG or PCI | 102 | 34 | Our definition of exacerbation of renal dysfunction was different from the known CI-AKI criteria such as the creatinine increasing rate over 25 % or delta creatinine value over 0.5 mg/dl by 48 h after the procedure |
| Yuan Y, 2018[94] | China, 2013-2015, | Asia | Elective PCI | 1,061 | 241 | Defined as an increase in SCr concentration ≥44.2 μmol/L or ≥25% above baseline within 72 h after exposure to contrast medium. |
| Yu-mei Gao, 2014[95] | China, 2005-2010 | Asia | Coronary angiography or percutaneous coronary intervention | 2,764 | 127 | Defined as an increase in SCr level C44.2 lmol/L or C25 % and simultaneously beyond the upper limit of normal value within 72 h following the intravascular administration of contrast media |
| Z. R. Xu, 2019[96] | China, 2015-2017 | Asia | Emergent PCI | 146 | 31 | CI-AKI, classically Defined by a relative (≥25%) or absolute (≥0.5 mg/dL; 44 μmol/L) increase in serum creatinine from baseline within 48 h after CM exposure, after ruling out other factors that could cause nephropathy |
| Susumu Yoshida, 2009[97] | Japan, 2002-2008 | Asia | Elective CAG or PCI | 431 | 36 | defined as an increase in the serum creatinine values of ≥25% or ≥0.5 mg/dl after contrast media exposure. |
| Carlo Budano, 2011[98] | Italy,  2007-2007 | Europe | Elective or emergency CAG | 755 | 120 | relative increase in SCr ≥25% over baseline within 48 hours |
| Mehmet Ball, 2015[99] | Turkey, 2013-2015 | Asia | PCI | 650 | 59 | defined by an increase in serum creatinine by >25% or 0.5 mg/dL within 72 h following contrast media exposure. |
| L. Barbieri, 2014[100] | Italy,  2007-2011 | Europe | CAG/PCI | 1950 | 251 | defined as an absolute ＞0.5 mg/dl or a relative ＞25% increase in the serum creatinine level at 24 or 48 h after the procedure |
| Davide Capodanno, 2016[101] | Italy,  2011-2011 | Europe | CAG/PCI | 706 | 96 | rise in serum creatinine ≥0.5 mg/dl and/or ≥ 25% increase in baseline serum creatinine |
| Muhammed Bora Demircelik，2015[102] | Turkey, 2009-2012 | Asia | Emergency PCI | 426 | 53 | defined as an increase in serum creatinine level of ≥ 0.5 mg/dl or 25% above baseline within 72 h after contrast administration |
| Dhaval Kolte, 2016[103] | USA,  2010-2014 | North America | PCI | 1162 | 78 | Defined as a relative rise in serum creatinine of ≥25%, or an absolute increase of ≥0.5 mg/dL, within 48 h of primary PCI |
| George Lazaros，2016[104] | Greece, 2006-2009 | Europe | Emergency PCI | 384 | 54 | CIN was defined as any of the following: increase in serum creatinine by ≥0.3 mg/dl within 48 hours; or increase in serum creatinine to ≥1.5 times compered to baseline values; or urine volume <0.5 ml/kg/h for 6 hours. |
| Yueping Li，2015[105] | China | Asia | Elective PCI | 719 | 37 | An increase in SCr of at least 25% or 44.2 mmol/L (0.5 mg/dL) within 3 days after CM administration in the absence of an alternative etiology |
| Atsushi Mizuno, 2014[106] | Japan | Asia | Emergency PCI | 102 | 10 | CI-AKI was defined as a >25% increase or an absolute increase in serum creatinine of 0.5 mg/dl within 3 days after percutaneous coronary intervention |
| Berk Özkaynak, 2014[107] | Turkey | Asia | CAG/PCI | 573 | 233 | AKIN |
| Dawlat Sany, 2014[108] | Egypt | Africa | CAG | 200 | 43 | Defined as rise in serum creatinine 48 h after contrast exposure of ＞0.5 mg/dL or increased425% compared to base line creatinine |
| Liwen Bao, 2017[109] | China | Asia | CAG/PCI | 163 | 14 | CIN was diagnosed when serum creatinine levels increased by 25% or 0.5 mg/dl after the use of contrasts |
| S. Kul, 2014[110] | Turkey | Asia | Emergency PCI | 314 | 38 | CI-AKI was defined as a relative increase in baseline serum Cr of greater than 25% and/or an absolute increase of 0.5 mg/ dl within 72 h after contrast administration |
| Maitri S. Pancholy, 2017[111] | USA | North America | PCI | 7529 | 151 | Defined as an increase in serum creatinine >0.5 mg/dl or>25% increase from baseline 48 to 72 hours after the procedure |
| Dong-Ho Shin, 2011[112] | Korea | Asia | CAG/PCI | 420 | 39 | Defined as an absolute increase in serum creatinine (SCr) >0.5 mg/dl (44.2 mol/L) or a relative increase >25% compared to baseline SCr |
| Maciej T. Wybraniec, 2017[113] | Poland | Europe | Emergency CAG/PCI | 95 | 9 | CI-AKI was defined as≥50% relative or≥0.3 mg/dL absolute increase of serum creatinine concentration at 48 h post-procedurally |
| Pablo Aguiar-Souto, 2010[114] | Australia | Oceania | PCI | 227 | 16 | CIN was defined as 24 h postprocedural increase of baseline creatinine levels ≥0.5 mg/dl (CIN05) or ≥25% (CIN25) |
| Giancarlo Marenzi, 2010[115] | Italy | Europe | PCI | 780 | 113 | Contrast-induced nephropathy was defined as an increase in serum creatinine N25% from baseline in the first 72 hours |
| Maryam PAKFETRA, 2010[116] | Iran | Asia | PCI | 290 | 45 | RIFLE criteria within 48 hours after contrast exposure |
| Fabio A. Sgura, 2010[117] | Italy | Europe | PCI | 891 | 64 | CIN was defined as a 0.5 mg/dL (44 mmol/L) increase in serum creatinine or 25% increase compared with baseline values within 48 hours of the procedure |
| Joshua M. Stolker, 2010[118] | USA | North America | CAG/PCI | 6358 | 823 | ＞0.3mg/dl absolute or 50% relative serum creatinine increase during 48 h after the procedure |
| Sergio Raposeiras-Roubın, 2013[119] | Spain | Europe | CAG | 940 | 54 | Contrast-induced nephropathy was defined as an increase in serum creatinine ≥0.5 mg/dL within 72 hr of exposure |
| Ahmet C¸ agrı ˘ Aykan, 2013[120] | Turkey | Asia | PCI | 402 | 131 | Contrast-induced nephropathy is defined as an increase of 25% or more in serum creatinine concentrations from baseline within 72 h following primary angioplasty |
| Hyang Mo Koo, 2013[121] | Korea | Asia | CAG | 735 | 64 | CIN was defined as an increase of ≥ 0.5 mg/dL or ≥ 25% in serum creatinine level during the 72 hours following PTCA |
| Toshijiro Aoki, 2018[122] | Japan | Asia | PCI | 443 | 88 | WRF was defined as an increase in serum creatinine levels ≥0.3 mg/dL above the admission value during the course of hospitalization |
| Yaron Arbel, 2019[123] | Canada | North America | PCI | 1692 | 208 | AKI was determined using the AKI network (AKIN) criteria and defined as an absolute increase of at least 0.3 mg/dl or a 50% relative increase |
| Lorenzo Azzalini, 2019[124] | Italy | Europe | PCI | 2660 | 312 | (AKIN) definition |
| Tufan Çınar, 2019[125] | Turkey | Asia | PCI | 1280 | 118 | Elevation of serum creatinine levels by 0.5 mg/dL or 25% that occurs within 72 h after the intravascular administration of contrast media |
| Fatih Gungoren, 2018[126] | Turkey | Asia | CAG/PCI | 269 | 46 | CIN was defined as a ≥0.5 mg/dL and/or a ≥25% increase in serum creatinine within 72 hour post-procedure |
| Jan Matějka, 2016[127] | Czech Republic | Europe | PCI | 202 | 25 | AKI was defined as an increase in serum creatinine ≥ 50% or 26.5 μmol/L (AKIN criteria) from the baseline within 48 h |
| Kenichiro Otsuka, 2018[128] | Japan | Asia | Emergency PCI | 334 | 71 | AKI was defined as an increase in serum creatinine of ≥ 0.3 mg/dl and/or ≥ 50% within 72 h after hospital admission |
| Johannes Schmucker, 2017[129] | Germany | Europe | CAG/PCI | 3810 | 690 | Acute kidney injury was graded from stage 0 to 3 following the Kidney-disease-improving-global outcomes criteria |
| Sinkovič A, 2019[130] | Slovenia | Europe | CAG/PCI | 245 | 34 | AKI was defined as a 1.5-fold increase in serum creatinine from baseline level within 24–48 hours |
| Ibrahim Yildiz, 2019[131] | Turkey | Asia | Emergency PCI | 768 | 93 | Contrast-induced nephropathy was defined as the impairment of renal function and was measured as either a relative increase of at least 25% or an absolute increase of at least 0.5 mg/dL in serum creatinine levels from baseline when there was no alternative etiology within 72 hours following primary angioplasty |
| Yong Wang, 2019[132] | China | Asia | Elective PCI | 239 | 39 | CIN was defined as a creatinine increase of at least 0.5 mg/dL or ≥ 25% from the baseline within 72 h following cardiac catheterization |
| Isil Uzunhasan, 2017[133] | Turkey | Asia | Emergency PCI | 1083 | 178 | Increase of ≥0.5 mg/dL and/or an increase of ≥25% of pre-percutaneous coronary intervention (PCI) to post-PCI serum creatinine levels within 48 to 72 hours after the procedure |
| Hitinder S. Gurm, 2011[134] | USA | North America | PCI | 58957 | 1470 | CIN was defined as impairment in renal function resulting in ＞0.5 mg/dl absolute increase in serum creatinine from baseline |

Abbreviations: CA-AKI, contrast-associated acute kidney injury; sCr, serum creatinine; AKI, acute kidney injury; RIFLE,

Risk, Injury, Failure, Loss, Endstage; pRIFLE, pediatric RIFLE; AKIN, Acute Kidney Injury Network; KDIGO, Kidney Disease Improving Global Outcomes; CAG, coronary angiography; PCI, percutaneous coronary intervention;

**Supplementary Table 2. Quality assessment of included studies**

| **Studies** | **Selection** | **Comparability** | **Outcome/ exposure** | **Total Score** |
| --- | --- | --- | --- | --- |
| Xun Hu, 2016 | **** | ** | *** | 9 |
| Saito Y, 2015 | *** | ** | *** | 8 |
| Mendi MA, 2016 | **** | ** | *** | 9 |
| Taeyoung Kong, 2017 | **** | ** | *** | 9 |
| Lucia Barbieri, 2016 | **** | ** | *** | 9 |
| Hyuck-Jun Yoon, 2013 | *** | ** | *** | 8 |
| Feldkamp T, 2017 | *** | * | *** | 7 |
| Han B, 2018 | *** | ** | ** | 7 |
| Qin YH, 2018 | **** | ** | *** | 9 |
| You ZB, 2018 | *** | * | *** | 7 |
| Yu-mei Gao, 2014 | **** | ** | *** | 9 |
| Hiroaki Watabe, 2014 | **** | ** | *** | 9 |
| Charanjit S, 2002 | **** | ** | *** | 9 |
| Giancarlo Marenzi, 2004 | **** | ** | ** | 8 |
| Ling Ji, 2015 | **** | ** | *** | 9 |
| Roxana Mehran, 2004 | **** | ** | *** | 9 |
| Ruey-Hsing Chou, 2016 | **** | ** | *** | 9 |
| Omer Toprak, 2007 | **** | ** | *** | 9 |
| Mitsuru Abe, 2009 | *** | ** | *** | 8 |
| Ali Amiri, 2018 | **** | * | *** | 8 |
| Ling Sun, 2018 | **** | ** | ** | 8 |
| Naikuan Fu, 2012 | *** | ** | *** | 8 |
| Warren K. Laskey, 2007 | **** | ** | *** | 9 |
| Denis F. Souza, 2015 | **** | * | *** | 8 |
| Sanjai Pattu Valappil, 2017 | **** | ** | *** | 9 |
| Javier A. Neyra, 2013 | **** | ** | *** | 9 |
| Min Young Rim, 2012 | **** | ** | *** | 9 |
| Kato K, 2008 | **** | ** | *** | 9 |
| Yuan Y, 2018 | *** | ** | *** | 8 |
| YousUKe Taniguchi, 2012 | *** | ** | *** | 8 |
| Hossein Nough, 2013 | **** | ** | ** | 8 |
| Pamela S. Reed, 2007 | *** | ** | *** | 8 |
| Nzgür Günebakmaz, 2013 | **** | ** | *** | 8 |
| Omer Celik, 2014 | **** | ** | *** | 9 |
| Thomas T. Tsai, 2014 | **** | ** | * | 7 |
| Murat Saritemur, 2014 | **** | ** | *** | 9 |
| Amal Abdel Ghani, 2009 | **** | ** | *** | 9 |
| Jae Hee Kim, 2014 | *** | ** | *** | 8 |
| Julio G. Peguero, 2013 | **** | ** | *** | 9 |
| Giuseppe Ando, 2013 | *** | ** | *** | 8 |
| Kosei Tanaga, 2012 | **** | ** | *** | 9 |
| Shao-Sung Huang, 2012 | **** | ** | *** | 9 |
| Salvatore Evola, 2012 | *** | ** | *** | 8 |
| Yijie Hu, 2013 | *** | ** | *** | 8 |
| David R. McIlroy, 2012 | **** | * | *** | 8 |
| Gustavo N. Araujo, 2017 | **** | ** | ** | 8 |
| Eyup Avci, 2017 | **** | * | *** | 8 |
| Toshiki Kuno, 2018 | **** | ** | *** | 9 |
| Silvia Esmeralda Pérez-Topete, 2016 | **** | * | ** | 7 |
| Xi-peng Sun, 2017 | **** | ** | *** | 9 |
| Florian Lüders, 2015 | *** | ** | *** | 8 |
| Taner Ulus, 2018 | *** | ** | *** | 8 |
| Chunrui Wang, 2017 | **** | ** | *** | 9 |
| Rui Wu, 2018 | *** | ** | *** | 8 |
| George Dangas, 2005 | **** | ** | *** | 9 |
| Praveen Kandula, 2010 | **** | ** | *** | 9 |
| Suhas S. Lele, 2013 | **** | ** | *** | 9 |
| Yalcin Velibey, 2016 | *** | ** | *** | 8 |
| Jae Yeong Cho, 2010 | **** | ** | *** | 9 |
| Victor SM, 2014 | **** | ** | *** | 9 |
| Dimitrios Tziakas, 2011 | **** | * | *** | 8 |
| Takeshi Senoo, 2010 | *** | ** | *** | 8 |
| Wang Ling, 2008 | **** | ** | *** | 9 |
| Wuhua Jiang, 2018 | *** | ** | *** | 8 |
| Y.R. Sedhai, 2017 | **** | ** | *** | 9 |
| Probal Roy, 2008 | *** | ** | *** | 8 |
| Omer Toprak, 2006 | **** | ** | *** | 9 |
| Serdar Farhan, 2015 | **** | * | *** | 8 |
| Irfan Sahin, 2013 | **** | ** | *** | 9 |
| Guoqiang Gu, 2017 | *** | * | *** | 7 |
| Kang Hyu Lee, 2010 | **** | ** | *** | 9 |
| Hakan Ucar, 2013 | **** | ** | *** | 9 |
| Masaomi Gohbara, 2017 | **** | * | ** | 7 |
| Eugenia Nikolsky, 2005 | **** | ** | *** | 9 |
| Jin Wi, 2012 | *** | ** | *** | 8 |
| Fei He, 2012 | **** | * | *** | 8 |
| Paulo Roberto Santos, 2015 | **** | ** | *** | 9 |
| Alparslan Kurtul, 2017 | **** | ** | *** | 9 |
| Samuel Goussot, 2015 | **** | ** | *** | 9 |
| Fei Gao, 2011 | **** | ** | *** | 9 |
| Rudolf Jarai, 2012 | **** | ** | *** | 9 |
| L. S. Nguyen, 2018 | **** | ** | *** | 9 |
| D. M. Leistner, 2018 | *** | ** | *** | 8 |
| J. A. Dodson, 2019 | **** | ** | * | 7 |
| Vojko Kanic, 2018 | **** | ** | *** | 9 |
| G. Cicek, 2017 | **** | ** | *** | 9 |
| C. Tang, 2019 | **** | * | *** | 8 |
| Ahmet Kaya, 2018 | **** | ** | *** | 9 |
| S. Sigirci, 2019 | **** | ** | *** | 9 |
| E. Izkhakov, 2019 | **** | ** | *** | 9 |
| Z. R. Xu, 2019 | *** | ** | *** | 8 |
| Felipe Hernández, 2009 | *** | ** | *** | 8 |
| Eric Chong, 2010 | **** | ** | *** | 9 |
| Feng Hua Ding, 2013 | **** | ** | *** | 9 |
| Abdurrezzak Börekçi, 2014 | **** | ** | *** | 9 |
| Jayakumar Sreenivasan, 2018 | **** | * | *** | 8 |
| Susumu Yoshida, 2009 | **** | ** | *** | 9 |
| Carlo Budano, 2011 | **** | ** | *** | 9 |
| Mehmet Ball, 2015 | **** | ** | *** | 9 |
| L. Barbieri, 2014 | **** | ** | *** | 9 |
| Davide Capodanno, 2016 | **** | ** | *** | 9 |
| Muhammed Bora Demircelik, 2015 | **** | ** | *** | 9 |
| Dhaval Kolte, 2016 | **** | ** | *** | 9 |
| George Lazaros, 2016 | **** | ** | *** | 9 |
| Yueping Li, 2015 | **** | ** | *** | 9 |
| Atsushi Mizuno, 2014 | **** | * | *** | 8 |
| Berk Özkaynak, 2014 | **** | ** | *** | 9 |
| Dawlat Sany, 2014 | **** | ** | *** | 9 |
| Liwen Bao, 2017 | **** | ** | *** | 9 |
| S. Kul, 2014 | **** | ** | *** | 9 |
| Maitri S. Pancholy, 2017 | **** | * | *** | 8 |
| Dong-Ho Shin, 2011 | **** | ** | *** | 9 |
| Maciej T. Wybraniec, 2017 | **** | * | *** | 8 |
| Pablo Aguiar-Souto, 2010 | **** | ** | *** | 9 |
| Giancarlo Marenzi, 2010 | **** |  | *** | 7 |
| Maryam PAKFETRAT, 2010 | **** | ** | *** | 9 |
| Fabio A. Sgura, 2010 | **** | * | *** | 8 |
| Joshua M. Stolker, 2010 | **** |  | *** | 7 |
| Sergio Raposeiras-Roubın, 2013 | **** |  | *** | 7 |
| Ahmet C¸ agrı ˘ Aykan, 2013 | **** | * | *** | 8 |
| Hyang Mo Koo, 2013 | **** | * | *** | 8 |
| Toshijiro Aoki, 2018 | **** | ** | *** | 9 |
| Yaron Arbel, 2019 | **** | ** | *** | 9 |
| Lorenzo Azzalini, 2019 | **** | ** | *** | 9 |
| Tufan Çınar, 2019 | **** | ** | *** | 9 |
| Fatih Gungoren, 2018 | **** | ** | *** | 9 |
| Jan Matějka, 2016 | **** | ** | *** | 9 |
| Kenichiro Otsuka, 2018 | **** | ** | *** | 9 |
| Johannes Schmucker, 2017 | **** | ** | *** | 9 |
| Sinkovič A, 2019 | **** | * | *** | 8 |
| Ibrahim Yildiz, 2019 | **** | * | *** | 8 |
| Yong Wang, 2019 | **** | ** | *** | 9 |
| Isil Uzunhasan, 2017 | **** | ** | *** | 9 |
| Hitinder S. Gurm, 2011 | **** | * | *** | 8 |

**Supplementary Table 3. Pooled incidence of CA-AKI and CA-AKI associated mortality according to different definitions.**

|  | Incidence (%) | Mortality (%) |
| --- | --- | --- |
| CA-AKI^#^ | 15.1 (95%CI: 12.9-17.4) | 20.6 (95%CI: 9.1-32.1) |
| CA-AKI^^^ | 16.0 (95%CI: 12.6-19.5) | 27.5 (95%CI: 7.8-47.2) |

CA-AKI^#^ was an increase in serum creatinine≥0.3 mg/dl or ≥50% from baseline within 72 hours.

CA-AKI^^^ were the criteria of acute kidney injury network (AKI stage 1, ≥0.3 mg/dl absolute or 1.5 to 2.0-fold relative increase in serum creatinine; AKI stage 2, >2- to 3-fold increase in serum creatinine; AKI stage 3, >3-fold increase in serum creatinine or serum creatinine >4.0 mg/dl with an acute increase of >0.5 mg/dl). or risk, injury, failure, loss of kidney function and end-stage kidney disease or Kidney-disease-improving-global outcomes.

**References**

1. Borekci A, Gur M, Turkoglu C, Cayli M, Selek S, Kaypakli O, Ucar H, Coskun M, Seker T, Koc M, Gokdeniz T, Gozukara MY (2015) Oxidative stress and paraoxonase 1 activity predict contrast-induced nephropathy in patients with ST-segment elevation myocardial infarction undergoing primary percutaneous coronary intervention. Angiology 66 (4):339-345. doi:10.1177/0003319714533588

2. Kaya A, Karatas A, Kaya Y, Dugeroglu H, Dereli S, Bayramoglu A (2018) A New and Simple Risk Predictor of Contrast-Induced Nephropathy in Patients Undergoing Primary Percutaneous Coronary Intervention: TIMI Risk Index. Cardiol Res Pract 2018:5908215. doi:10.1155/2018/5908215

3. Amiri A, Ghanavati R, Riahi Beni H, Sezavar SH, Sheykhvatan M, Arab M (2018) Metabolic Syndrome and the Iodine-Dose/Creatinine Clearance Ratio as Determinants of Contrast-Induced Acute Kidney Injury. Cardiorenal medicine 8 (3):217-227. doi:10.1159/000488374

4. Kurtul A, Yarlioglues M, Duran M (2017) Predictive Value of CHA2DS2-VASC Score for Contrast-Induced Nephropathy After Percutaneous Coronary Intervention for Acute Coronary Syndrome. The American journal of cardiology 119 (6):819-825. doi:10.1016/j.amjcard.2016.11.033

5. Ghani AA, Tohamy KY (2009) Risk score for contrast induced nephropathy following percutaneous coronary intervention. Saudi journal of kidney diseases and transplantation : an official publication of the Saudi Center for Organ Transplantation, Saudi Arabia 20 (2):240-245

6. Tang C, Hou J, Yan G, Qiao Y, Wang D, Zhu B, Liu B, Luo E, Nawabi AQ, Chen L (2019) Effects of Serum Cytochrome c on Contrast-Induced Nephropathy in Patients with ST-Elevation Myocardial Infarction Undergoing Percutaneous Coronary Intervention. BioMed research international 2019:9357203. doi:10.1155/2019/9357203

7. Rihal CS, Textor SC, Grill DE, Berger PB, Ting HH, Best PJ, Singh M, Bell MR, Barsness GW, Mathew V, Garratt KN, Holmes DR, Jr. (2002) Incidence and prognostic importance of acute renal failure after percutaneous coronary intervention. Circulation 105 (19):2259-2264. doi:10.1161/01.cir.0000016043.87291.33

8. Wang C, Ma S, Deng B, Lu J, Shen W, Jin B, Shi H, Ding F (2017) The predictive value of the product of contrast medium volume and urinary albumin/creatinine ratio in contrast-induced acute kidney injury. Renal failure 39 (1):555-560. doi:10.1080/0886022x.2017.1349673

9. Leistner DM, Munch C, Steiner J, Erbay A, Riedel M, Gebhard C, Lauten A, Landmesser U, Stahli BE (2018) Impact of acute kidney injury in elderly (>/=80 years) patients undergoing percutaneous coronary intervention. Journal of interventional cardiology 31 (6):792-798. doi:10.1111/joic.12547

10. McIlroy DR, Epi MC, Argenziano M, Farkas D, Umann T (2012) Acute kidney injury after cardiac surgery: does the time interval from contrast administration to surgery matter? Journal of cardiothoracic and vascular anesthesia 26 (5):804-812. doi:10.1053/j.jvca.2012.02.013

11. Souza DF, Reis SS, Botelho RV, Ferreira-Filho SR (2015) Relative and absolute changes in urinary neutrophil gelatinase-associated lipocalin and correlation with small increases in serum creatinine levels after coronary angiography: an observational study. Nephron 129 (2):84-90. doi:10.1159/000368413

12. Tziakas D, Chalikias G, Stakos D, Apostolakis S, Adina T, Kikas P, Alexoudis A, Passadakis P, Thodis E, Vargemezis V, Konstantinides S (2013) Development of an easily applicable risk score model for contrast-induced nephropathy prediction after percutaneous coronary intervention: a novel approach tailored to current practice. Int J Cardiol 163 (1):46-55. doi:10.1016/j.ijcard.2011.05.079

13. Izkhakov E, Rozenbaum Z, Margolis G, Khoury S, Keren G, Shacham Y (2019) Prolonged Hyperglycemia and Renal Failure after Primary Percutaneous Coronary Intervention. Cardiorenal medicine 9 (2):92-99. doi:10.1159/000495704

14. Chong E, Poh KK, Shen L, Chai P, Tan HC (2009) Diabetic patients with normal baseline renal function are at increased risk of developing contrast-induced nephropathy post-percutaneous coronary intervention. Singapore medical journal 50 (3):250-254

15. Nikolsky E, Mehran R, Lasic Z, Mintz GS, Lansky AJ, Na Y, Pocock S, Negoita M, Moussa I, Stone GW, Moses JW, Leon MB, Dangas G (2005) Low hematocrit predicts contrast-induced nephropathy after percutaneous coronary interventions. Kidney international 67 (2):706-713. doi:10.1111/j.1523-1755.2005.67131.x

16. Avci E, Yildirim T, Kadi H (2017) Contrast-Induced Nephropathy Is Less Common in Patients with Good Coronary Collateral Circulation. Cardiorenal medicine 7 (4):316-323. doi:10.1159/000479013

17. Gao F, Zhou YJ, Zhu X, Wang ZJ, Yang SW, Shen H (2011) C-reactive protein and the risk of contrast-induced acute kidney injury in patients undergoing percutaneous coronary intervention. American journal of nephrology 34 (3):203-210. doi:10.1159/000329534

18. He F, Zhang J, Lu ZQ, Gao QL, Sha DJ, Pei LG, Fan GF (2012) Risk factors and outcomes of acute kidney injury after intracoronary stent implantation. World journal of emergency medicine 3 (3):197-201. doi:10.5847/wjem.j.1920-8642.2012.03.007

19. Feldkamp T, Luedemann M, Spehlmann ME, Freitag-Wolf S, Gaensbacher J, Schulte K, Bajrovic A, Hinzmann D, Hippe HJ, Kunzendorf U, Frey N, Luedde M (2018) Radial access protects from contrast media induced nephropathy after cardiac catheterization procedures. Clinical research in cardiology : official journal of the German Cardiac Society 107 (2):148-157. doi:10.1007/s00392-017-1166-2

20. Hernandez F, Mora L, Garcia-Tejada J, Velazquez M, Gomez-Blazquez I, Bastante T, Albarran A, Andreu J, Tascon J (2009) Comparison of iodixanol and ioversol for the prevention of contrast-induced nephropathy in diabetic patients after coronary angiography or angioplasty. Revista espanola de cardiologia 62 (12):1373-1380. doi:10.1016/s1885-5857(09)73531-5

21. Ding FH, Lu L, Zhang RY, Zhu TQ, Pu LJ, Zhang Q, Chen QJ, Hu J, Yang ZK, Shen WF (2013) Impact of elevated serum glycated albumin levels on contrast-induced acute kidney injury in diabetic patients with moderate to severe renal insufficiency undergoing coronary angiography. International journal of cardiology 167 (2):369-373. doi:10.1016/j.ijcard.2011.12.101

22. Luders F, Meyborg M, Malyar N, Reinecke H (2015) The Preinterventional Cystatin-Creatinine-Ratio: A Prognostic Marker for Contrast Medium-Induced Acute Kidney Injury and Long-Term All-Cause Mortality. Nephron 131 (1):59-65. doi:10.1159/000438755

23. Cicek G, Yildirim E (2018) CHA2DS2-VASc score predicts contrast-induced nephropathy in patients with ST-segment elevation myocardial infarction, who have undergone primary percutaneous coronary intervention. Kardiologia polska 76 (1):91-98. doi:10.5603/KP.a2017.0177

24. Dangas G, Iakovou I, Nikolsky E, Aymong ED, Mintz GS, Kipshidze NN, Lansky AJ, Moussa I, Stone GW, Moses JW, Leon MB, Mehran R (2005) Contrast-induced nephropathy after percutaneous coronary interventions in relation to chronic kidney disease and hemodynamic variables. The American journal of cardiology 95 (1):13-19. doi:10.1016/j.amjcard.2004.08.056

25. Marenzi G, Lauri G, Assanelli E, Campodonico J, De Metrio M, Marana I, Grazi M, Veglia F, Bartorelli AL (2004) Contrast-induced nephropathy in patients undergoing primary angioplasty for acute myocardial infarction. Journal of the American College of Cardiology 44 (9):1780-1785. doi:10.1016/j.jacc.2004.07.043

26. Ando G, Morabito G, de Gregorio C, Trio O, Saporito F, Oreto G (2013) Age, glomerular filtration rate, ejection fraction, and the AGEF score predict contrast-induced nephropathy in patients with acute myocardial infarction undergoing primary percutaneous coronary intervention. Catheterization and cardiovascular interventions : official journal of the Society for Cardiac Angiography & Interventions 82 (6):878-885. doi:10.1002/ccd.25023

27. Gu G, Xing H, Zhou Y, Cui W (2018) Inverse correlation between left ventricular end-diastolic pressure and contrast-induced nephropathy in patients undergoing percutaneous coronary intervention. Clinical and experimental nephrology 22 (4):808-814. doi:10.1007/s10157-017-1514-6

28. Araujo GN, Pivatto Junior F, Fuhr B, Cassol EP, Machado GP, Valle FH, Bergoli LC, Wainstein RV, Polanczyk CA, Wainstein MV (2018) Simplifying contrast-induced acute kidney injury prediction after primary percutaneous coronary intervention: the age, creatinine and ejection fraction score. Cardiovascular intervention and therapeutics 33 (3):224-231. doi:10.1007/s12928-017-0472-y

29. Ucar H, Gur M, Yildirim A, Borekci A, Gozukara MY, Seker T, Kaypakli O, Turkoglu C, Ozaltun B, Akyol S, Harbalioglu H, Sahin DY, Elbasan Z, Cayli M (2014) Increased aortic stiffness predicts contrast-induced nephropathy in patients with stable coronary artery disease undergoing percutaneous coronary intervention. Angiology 65 (9):806-811. doi:10.1177/0003319713504126

30. Han B, Li Y, Dong Z, Wan Q, Shen H, Li J, Wei M, Shen C (2018) Diastolic dysfunction predicts the risk of contrast-induced nephropathy and outcome post-emergency percutaneous coronary intervention in AMI patients with preserved ejection fraction. Heart Vessels 33 (10):1149-1158. doi:10.1007/s00380-018-1165-x

31. Watabe H, Sato A, Hoshi T, Takeyasu N, Abe D, Akiyama D, Kakefuda Y, Nishina H, Noguchi Y, Aonuma K (2014) Association of contrast-induced acute kidney injury with long-term cardiovascular events in acute coronary syndrome patients with chronic kidney disease undergoing emergent percutaneous coronary intervention. International journal of cardiology 174 (1):57-63. doi:10.1016/j.ijcard.2014.03.146

32. Nough H, Eghbal F, Soltani M, Nejafi F, Falahzadeh H, Fazel H, Sheikhvatan M (2013) Incidence and Main Determinants of Contrast-Induced Nephropathy following Coronary Angiography or Subsequent Balloon Angioplasty. Cardiorenal medicine 3 (2):128-135. doi:10.1159/000351981

33. Yoon HJ, Kim H, Lee JP, Choi SW, Cho HO, Shin HW, Park HS, Cho YK, Nam CW, Hur SH, Kim YN, Kim KB (2013) The efficacy of the cystatin C based glomerular filtration rate in the estimation of safe contrast media volume. Korean circulation journal 43 (9):622-627. doi:10.4070/kcj.2013.43.9.622

34. Sahin I, Gungor B, Can MM, Avci, II, Guler GB, Okuyan E, Biter H, Yildiz SS, Ayca B, Satilmis S, Dinckal MH (2014) Lower blood vitamin D levels are associated with an increased incidence of contrast-induced nephropathy in patients undergoing coronary angiography. The Canadian journal of cardiology 30 (4):428-433. doi:10.1016/j.cjca.2013.12.029

35. Dodson JA, Hajduk A, Curtis J, Geda M, Krumholz HM, Song X, Tsang S, Blaum C, Miller P, Parikh CR, Chaudhry SI (2019) Acute kidney injury among older patients undergoing coronary angiography for acute myocardial infarction: The SILVER-AMI Study. The American journal of medicine. doi:10.1016/j.amjmed.2019.05.022

36. Kim JH, Lee JH, Jang SY, Park SH, Bae MH, Yang DH, Park HS, Cho Y, Chae SC (2014) Prognostic value of early acute kidney injury after primary percutaneous coronary intervention in patients with ST-segment elevation myocardial infarction. The American journal of cardiology 114 (8):1174-1178. doi:10.1016/j.amjcard.2014.07.039

37. Cho JY, Jeong MH, Hwan Park S, Kim IS, Park KH, Sim DS, Yoon NS, Yoon HJ, Park HW, Hong YJ, Kim JH, Ahn Y, Cho JG, Park JC, Kang JC (2010) Effect of contrast-induced nephropathy on cardiac outcomes after use of nonionic isosmolar contrast media during coronary procedure. Journal of cardiology 56 (3):300-306. doi:10.1016/j.jjcc.2010.07.002

38. Neyra JA, Shah S, Mooney R, Jacobsen G, Yee J, Novak JE (2013) Contrast-induced acute kidney injury following coronary angiography: a cohort study of hospitalized patients with or without chronic kidney disease. Nephrology, dialysis, transplantation : official publication of the European Dialysis and Transplant Association - European Renal Association 28 (6):1463-1471. doi:10.1093/ndt/gft082

39. Sreenivasan J, Zhuo M, Khan MS, Li H, Fugar S, Desai P, Yadav N (2018) Anemia (Hemoglobin </= 13 g/dL) as a Risk Factor for Contrast-Induced Acute Kidney Injury Following Coronary Angiography. Am J Cardiol 122 (6):961-965. doi:10.1016/j.amjcard.2018.06.012

40. Wi J, Ko YG, Shin DH, Kim JS, Kim BK, Choi D, Ha JW, Hong MK, Jang Y (2013) Prediction of Contrast-Induced Nephropathy With Persistent Renal Dysfunction and Adverse Long-term Outcomes in Patients With Acute Myocardial Infarction Using the Mehran Risk Score. Clinical cardiology 36 (1):46-53. doi:10.1002/clc.22060

41. Peguero JG, Cornielle V, Gomez SI, Issa OM, Heimowitz TB, Santana O, Goldszer RC, Lamas GA (2014) The use of nitrates in the prevention of contrast-induced nephropathy in patients hospitalized after undergoing percutaneous coronary intervention. Journal of cardiovascular pharmacology and therapeutics 19 (3):310-314. doi:10.1177/1074248413515077

42. Lee KH, Lee SR, Kang KP, Kim HJ, Lee SH, Rhee KS, Chae JK, Kim WH, Ko JK (2010) Periprocedural hemoglobin drop and contrast-induced nephropathy in percutaneous coronary intervention patients. Korean circulation journal 40 (2):68-73. doi:10.4070/kcj.2010.40.2.68

43. Kato K, Sato N, Yamamoto T, Iwasaki YK, Tanaka K, Mizuno K (2008) Valuable markers for contrast-induced nephropathy in patients undergoing cardiac catheterization. Circulation journal : official journal of the Japanese Circulation Society 72 (9):1499-1505. doi:10.1253/circj.cj-07-1006

44. Tanaga K, Tarao K, Nakamura Y, Inoue T, Jo K, Ishikawa T, Miyazaki A (2012) Percutaneous coronary intervention causes increase of serum cystatin C concentration even in the patients with a low risk of contrast-induced nephropathy. Cardiovascular intervention and therapeutics 27 (3):168-173. doi:10.1007/s12928-012-0106-3

45. Nguyen LS, Spagnoli V, Kerneis M, Hauguel-Moreau M, Barthelemy O, Collet JP, Montalescot G, Silvain J (2019) Evaluation of neutrophil gelatinase-associated lipocalin and cystatin C as biomarkers of acute kidney injury after ST-segment elevation myocardial infarction treated by percutaneous coronary intervention. Archives of cardiovascular diseases 112 (3):180-186. doi:10.1016/j.acvd.2018.11.006

46. Ji L, Su X, Qin W, Mi X, Liu F, Tang X, Li Z, Yang L (2015) Novel risk score of contrast-induced nephropathy after percutaneous coronary intervention. Nephrology (Carlton, Vic) 20 (8):544-551. doi:10.1111/nep.12429

47. Sun L, Zhou X, Jiang J, Zang X, Chen X, Li H, Cao H, Wang Q (2018) Growth differentiation factor-15 levels and the risk of contrast induced acute kidney injury in acute myocardial infarction patients treated invasively: A propensity-score match analysis. PloS one 13 (3):e0194152. doi:10.1371/journal.pone.0194152

48. Barbieri L, Verdoia M, Nardin M, Marino P, Suryapranata H, De Luca G (2017) Gender Difference in the Risk of Contrast-Induced Nephropathy in Patients Undergoing Coronary Angiography or Percutaneous Coronary Intervention. Angiology 68 (6):542-546. doi:10.1177/0003319716669429

49. Gohbara M, Hayakawa A, Akazawa Y, Furihata S, Kondo A, Fukushima Y, Tomari S, Endo T, Kimura K, Tamura K (2017) Association Between Acidosis Soon After Reperfusion and Contrast-Induced Nephropathy in Patients With a First-Time ST-Segment Elevation Myocardial Infarction. Journal of the American Heart Association 6 (8). doi:10.1161/jaha.117.006380

50. Mendi MA, Afsar B, Oksuz F, Turak O, Yayla C, Ozcan F, Johnson RJ, Kanbay M (2017) Uric Acid is a Useful Tool to Predict Contrast-Induced Nephropathy. Angiology 68 (7):627-632. doi:10.1177/0003319716639187

51. Rim MY, Ro H, Kang WC, Kim AJ, Park H, Chang JH, Lee HH, Chung W, Jung JY (2012) The effect of renin-angiotensin-aldosterone system blockade on contrast-induced acute kidney injury: a propensity-matched study. American journal of kidney diseases : the official journal of the National Kidney Foundation 60 (4):576-582. doi:10.1053/j.ajkd.2012.04.017

52. Abe M, Kimura T, Morimoto T, Furukawa Y, Kita T (2009) Incidence of and risk factors for contrast-induced nephropathy after cardiac catheterization in Japanese patients. Circulation journal : official journal of the Japanese Circulation Society 73 (8):1518-1522. doi:10.1253/circj.cj-08-0784

53. Saritemur M, Turkeli M, Kalkan K, Tanboga IH, Aksakal E (2014) Relation of uric acid and contrast-induced nephropathy in patients undergoing primary percutaneous coronary intervention in the ED. The American journal of emergency medicine 32 (2):119-123. doi:10.1016/j.ajem.2013.10.011

54. Fu N, Li X, Yang S, Chen Y, Li Q, Jin D, Cong H (2013) Risk score for the prediction of contrast-induced nephropathy in elderly patients undergoing percutaneous coronary intervention. Angiology 64 (3):188-194. doi:10.1177/0003319712467224

55. Celik O, Ozturk D, Akin F, Ayca B, Yalcin AA, Erturk M, Biyik I, Ayaz A, Akturk IF, Enhos A, Aslan S (2015) Association Between Contrast Media Volume-Glomerular Filtration Rate Ratio and Contrast-Induced Acute Kidney Injury After Primary Percutaneous Coronary Intervention. Angiology 66 (6):519-524. doi:10.1177/0003319714542277

56. Toprak O, Cirit M, Esi E, Postaci N, Yesil M, Bayata S (2006) Hyperuricemia as a risk factor for contrast-induced nephropathy in patients with chronic kidney disease. Catheterization and cardiovascular interventions : official journal of the Society for Cardiac Angiography & Interventions 67 (2):227-235. doi:10.1002/ccd.20598

57. Toprak O, Cirit M, Yesil M, Bayata S, Tanrisev M, Varol U, Ersoy R, Esi E (2007) Impact of diabetic and pre-diabetic state on development of contrast-induced nephropathy in patients with chronic kidney disease. Nephrology, dialysis, transplantation : official publication of the European Dialysis and Transplant Association - European Renal Association 22 (3):819-826. doi:10.1093/ndt/gfl636

58. Gunebakmaz O, Duran M, Karakaya E, Tanrikulu E, Akpek M, Ergin A, Kaya MG (2013) Increased serum asymmetric dimethylarginine level is an independent predictor of contrast-induced nephropathy. Turk Kardiyoloji Dernegi arsivi : Turk Kardiyoloji Derneginin yayin organidir 41 (7):581-588. doi:10.5543/tkda.2013.88403

59. Reed PS, Dixon SR, Boura JA, O'Neill WW, Kahn JK (2007) Comparison of the usefulness of gadodiamide and iodine mixture versus iodinated contrast alone for prevention of contrast-induced nephropathy in patients with chronic kidney disease undergoing coronary angiography. The American journal of cardiology 100 (7):1090-1093. doi:10.1016/j.amjcard.2007.04.055

60. Santos PR, Carneiro Neto JD, Arcanjo FP, Carneiro JK, Carneiro RC, do Amaral CL (2015) Contrast-induced nephropathy after primary angioplasty for acute myocardial infarction. Jornal brasileiro de nefrologia : 'orgao oficial de Sociedades Brasileira e Latino-Americana de Nefrologia 37 (4):439-445. doi:10.5935/0101-2800.20150070

61. Kandula P, Shah R, Singh N, Markwell SJ, Bhensdadia N, Navaneethan SD (2010) Statins for prevention of contrast-induced nephropathy in patients undergoing non-emergent percutaneous coronary intervention. Nephrology (Carlton, Vic) 15 (2):165-170. doi:10.1111/j.1440-1797.2009.01204.x

62. Roy P, Raya V, Okabe T, Pinto Slottow TL, Steinberg DH, Smith K, Xue Z, Satler LF, Kent KM, Suddath WO, Pichard AD, Lindsay J, Waksman R (2008) Incidence, predictors, and outcomes of post-percutaneous coronary intervention nephropathy in patients with diabetes mellitus and normal baseline serum creatinine levels. The American journal of cardiology 101 (11):1544-1549. doi:10.1016/j.amjcard.2008.02.035

63. Qin YH, Yan GL, Ma CL, Tang CC, Ma GS (2018) Effects of hyperglycaemia and elevated glycosylated haemoglobin on contrast-induced nephropathy after coronary angiography. Experimental and therapeutic medicine 16 (1):377-383. doi:10.3892/etm.2018.6183

64. Mehran R, Aymong ED, Nikolsky E, Lasic Z, Iakovou I, Fahy M, Mintz GS, Lansky AJ, Moses JW, Stone GW, Leon MB, Dangas G (2004) A simple risk score for prediction of contrast-induced nephropathy after percutaneous coronary intervention: development and initial validation. J Am Coll Cardiol 44 (7):1393-1399. doi:10.1016/j.jacc.2004.06.068

65. Jarai R, Dangas G, Huber K, Xu K, Brodie BR, Witzenbichler B, Metzger DC, Radke PW, Yu J, Claessen BE, Genereux P, Mehran R, Stone GW (2012) B-type natriuretic peptide and risk of contrast-induced acute kidney injury in acute ST-segment-elevation myocardial infarction: a substudy from the HORIZONS-AMI trial. Circulation Cardiovascular interventions 5 (6):813-820. doi:10.1161/circinterventions.112.972356

66. Chou RH, Huang PH, Hsu CY, Leu HB, Huang SS, Huang CC, Chen JW, Lin SJ (2016) CHADS2 score predicts risk of contrast-induced nephropathy in stable coronary artery disease patients undergoing percutaneous coronary interventions. Journal of the Formosan Medical Association = Taiwan yi zhi 115 (7):501-509. doi:10.1016/j.jfma.2015.12.008

67. Wu R, Kong Y, Yin J, Liang R, Lu Z, Wang N, Zhao Q, Zhou Y, Yan C, Wang F, Liang M (2018) Antithrombin is a Novel Predictor for Contrast Induced Nephropathy After Coronary Angiography. Kidney & blood pressure research 43 (1):170-180. doi:10.1159/000487499

68. Sigirci S, Keskin K, Yildiz SS, Cetinkal G, Gurdal A, Kilci H, Tezcan M, Kilickesmez KO (2019) Can Thrombus Burden Predict Contrast-Induced Nephropathy in Patients With ST-Segment Elevation Myocardial Infarction? Angiology 70 (7):642-648. doi:10.1177/0003319718822638

69. Saito Y, Watanabe M, Aonuma K, Hirayama A, Tamaki N, Tsutsui H, Murohara T, Ogawa H, Akasaka T, Yoshimura M, Sato A, Takayama T, Sakakibara M, Suzuki S, Ishigami K, Onoue K (2015) Proteinuria and Reduced Estimated Glomerular Filtration Rate Are Independent Risk Factors for Contrast-Induced Nephropathy After Cardiac Catheterization. Circulation journal : official journal of the Japanese Circulation Society 79 (7):1624-1630. doi:10.1253/circj.CJ-14-1345

70. Evola S, Lunetta M, Macaione F, Fonte G, Milana G, Corrado E, Bonura F, Novo G, Hoffmann E, Novo S (2012) Risk factors for contrast induced nephropathy: a study among Italian patients. Indian heart journal 64 (5):484-491. doi:10.1016/j.ihj.2012.07.007

71. Goussot S, Mousson C, Guenancia C, Stamboul K, Brunel P, Brunet D, Touzery C, Cottin Y, Zeller M (2015) N-Terminal Fragment of Pro B-type Natriuretic Peptide as a Marker of Contrast-Induced Nephropathy After Primary Percutaneous Coronary Intervention for ST-Segment Elevation Myocardial Infarction. The American journal of cardiology 116 (6):865-871. doi:10.1016/j.amjcard.2015.06.007

72. Valappil SP, Kunjukrishnapillai S, Iype M, Koshy AG, Viswanathan S, Gupta PN, Velayudhan RV, Ali FM (2018) Predictors of contrast induced nephropathy and the applicability of the Mehran risk score in high risk patients undergoing coronary angioplasty-A study from a tertiary care center in South India. Indian heart journal 70 (3):399-404. doi:10.1016/j.ihj.2017.08.018

73. Farhan S, Vogel B, Tentzeris I, Jarai R, Freynhofer MK, Smetana P, Egger F, Kautzky-Willer A, Huber K (2016) Contrast induced acute kidney injury in acute coronary syndrome patients: A single centre experience. Eur Heart J Acute Cardiovasc Care 5 (1):55-61. doi:10.1177/2048872615574707

74. Huang SS, Huang PH, Leu HB, Wu TC, Lin SJ, Chen JW (2013) Association of central pulse pressure with contrast-induced nephropathy and clinical outcomes in patients undergoing coronary intervention. Journal of hypertension 31 (11):2187-2194. doi:10.1097/HJH.0b013e3283641023

75. Pérez-Topete SE, Miranda-Aquino T, Gasca-Luna K, Guerra-Villa MN, Elizondo-Adamchik HE (2016) Contrast-induced nephropathy in patients undergoing percutaneous coronary intervention. Revista Mexicana de Cardiología 27 (2):64-70

76. Lele SS, Mukhopadhyay BN, Mardikar MM, Patel TA, Vasavada AK, Banker DN, Kapasi KD, Chauhan VC, Chawla KC, Raju SR, Hiremath SS, Chinchole SS, Rajapurkar MM (2013) Impact of catalytic iron on mortality in patients with acute coronary syndrome exposed to iodinated radiocontrast-The Iscom Study. American heart journal 165 (5):744-751. doi:10.1016/j.ahj.2013.02.016

77. Kong T, Park YS, Lee HS, Kim S, Han S, Eun CH, Lee JW, You JS, Chung HS, Park I, Chung SP (2018) A Delta Neutrophil Index for the Prediction of Contrast-Induced Nephropathy in Patients With St-Elevation Myocardial Infarction Followed By Percutaneous Coronary Intervention. Shock (Augusta, Ga) 49 (3):317-325. doi:10.1097/shk.0000000000000957

78. Senoo T, Motohiro M, Kamihata H, Yamamoto S, Isono T, Manabe K, Sakuma T, Yoshida S, Sutani Y, Iwasaka T (2010) Contrast-induced nephropathy in patients undergoing emergency percutaneous coronary intervention for acute coronary syndrome. The American journal of cardiology 105 (5):624-628. doi:10.1016/j.amjcard.2009.10.044

79. Ulus T, Isgandarov K, Yilmaz AS, Uysal S, Vasi I, Dural M, Mutlu F (2018) Monocyte to High-Density Lipoprotein Ratio Predicts Contrast-Induced Nephropathy in Patients With Acute Coronary Syndrome. Angiology 69 (10):909-916. doi:10.1177/0003319718760916

80. Tsai TT, Patel UD, Chang TI, Kennedy KF, Masoudi FA, Matheny ME, Kosiborod M, Amin AP, Weintraub WS, Curtis JP, Messenger JC, Rumsfeld JS, Spertus JA (2014) Validated contemporary risk model of acute kidney injury in patients undergoing percutaneous coronary interventions: insights from the National Cardiovascular Data Registry Cath-PCI Registry. Journal of the American Heart Association 3 (6):e001380. doi:10.1161/jaha.114.001380

81. Kuno T, Numasawa Y, Sawano M, Katsuki T, Kodaira M, Ueda I, Suzuki M, Noma S, Negishi K, Ishikawa S, Miyata H, Fukuda K, Kohsaka S (2018) Effects of body habitus on contrast-induced acute kidney injury after percutaneous coronary intervention. PLoS One 13 (9):e0203352. doi:10.1371/journal.pone.0203352

82. Victor SM, Gnanaraj A, S V, Deshmukh R, Kandasamy M, Janakiraman E, Pandurangi UM, Latchumanadhas K, Abraham G, Mullasari AS (2014) Risk scoring system to predict contrast induced nephropathy following percutaneous coronary intervention. Indian heart journal 66 (5):517-524. doi:10.1016/j.ihj.2014.05.025

83. Kanic V, Kompara G, Suran D, Tapajner A, Naji FH, Sinkovic A (2019) Acute kidney injury in patients with myocardial infarction undergoing percutaneous coronary intervention using radial versus femoral access. BMC nephrology 20 (1):28. doi:10.1186/s12882-019-1210-8

84. Ling W, Zhaohui N, Ben H, Leyi G, Jianping L, Huili D, Jiaqi Q (2008) Urinary IL-18 and NGAL as early predictive biomarkers in contrast-induced nephropathy after coronary angiography. Nephron Clinical practice 108 (3):c176-181. doi:10.1159/000117814

85. Laskey WK, Jenkins C, Selzer F, Marroquin OC, Wilensky RL, Glaser R, Cohen HA, Holmes DR, Jr. (2007) Volume-to-creatinine clearance ratio: a pharmacokinetically based risk factor for prediction of early creatinine increase after percutaneous coronary intervention. Journal of the American College of Cardiology 50 (7):584-590. doi:10.1016/j.jacc.2007.03.058

86. Jiang W, Shen B, Wang Y, Xu J, Luo Z, Ding X, Teng J (2019) Potentially Modifiable Predictors for Renal Replacement Therapy in Patients with Cardiac Surgery Associated-Acute Kidney Injury: a Propensity Score-Matched Case-Control Study. Brazilian journal of cardiovascular surgery 34 (1):33-40. doi:10.21470/1678-9741-2018-0206

87. Sun XP, Li J, Zhu WW, Li DB, Chen H, Li HW, Chen WM, Hua Q (2018) Platelet to Lymphocyte Ratio Predicts Contrast-Induced Nephropathy in Patients With ST-Segment Elevation Myocardial Infarction Undergoing Primary Percutaneous Coronary Intervention. Angiology 69 (1):71-78. doi:10.1177/0003319717707410

88. Hu X, Zhuang XD, Li Y, Li FF, Guo Y, Du ZM, Liao XX (2017) A Nomogram to Predict Contrast Induced Nephropathy in Patients Undergoing Percutaneous Coronary Intervention. International heart journal 58 (2):191-196. doi:10.1536/ihj.16-213

89. Sedhai YR, Golamari R, Timalsina S, Basnyat S, Koirala A, Asija A, Choksi T, Kushwah A, Geovorgyan D, Dar T, Borikar M, Ahangar W, Alukal J, Zia S, Missri J (2017) Contrast-Induced Nephropathy After Cardiac Catheterization: Culprits, Consequences and Predictors. The American journal of the medical sciences 354 (5):462-466. doi:10.1016/j.amjms.2017.05.010

90. Velibey Y, Oz A, Tanik O, Guvenc TS, Kalenderoglu K, Gumusdag A, Akdeniz E, Bozbay M, Tekkesin AI, Guzelburc O, Hayiroglu MI, Alper AT, Ugur M, Eren M (2017) Platelet-to-Lymphocyte Ratio Predicts Contrast-Induced Acute Kidney Injury in Patients With ST-Segment Elevation Myocardial Infarction Undergoing Primary Percutaneous Coronary Intervention. Angiology 68 (5):419-427. doi:10.1177/0003319716660244

91. Hu Y, Li Z, Chen J, Shen C, Song Y, Zhong Q (2013) Risk factors for acute kidney injury in patients undergoing same admission coronary angiography and valve replacement. Journal of cardiac surgery 28 (6):627-631. doi:10.1111/jocs.12222

92. You ZB, Lin KY, Zheng WP, Lin CJ, Lin F, Guo TL, Zhu PL, Guo YS (2018) Association of prealbumin levels with contrast-induced acute kidney injury in elderly patients with elective percutaneous coronary intervention. Clinical interventions in aging 13:641-649. doi:10.2147/cia.s162764

93. Taniguchi Y, Sakakura K, Wada H, Sugawara Y, Funayama H, Kubo N, Momomura S, Ako J (2013) Contrast induced exacerbation of renal dysfunction in the advanced chronic kidney disease. Cardiovascular intervention and therapeutics 28 (2):157-161. doi:10.1007/s12928-012-0143-y

94. Yuan Y, Qiu H, Song L, Hu X, Luo T, Zhao X, Zhang J, Wu Y, Qiao S, Yang Y, Gao R (2018) A New Risk Factor Profile for Contrast-Induced Acute Kidney Injury in Patients Who Underwent an Emergency Percutaneous Coronary Intervention. Angiology 69 (6):523-531. doi:10.1177/0003319717736157

95. Gao YM, Li D, Cheng H, Chen YP (2014) Derivation and validation of a risk score for contrast-induced nephropathy after cardiac catheterization in Chinese patients. Clinical and experimental nephrology 18 (6):892-898. doi:10.1007/s10157-014-0942-9

96. Xu ZR, Chen J, Liu YH, Liu Y, Tan N (2019) The predictive value of the renal resistive index for contrast-induced nephropathy in patients with acute coronary syndrome. BMC cardiovascular disorders 19 (1):36. doi:10.1186/s12872-019-1017-3

97. Yoshida S, Kamihata H, Nakamura S, Senoo T, Manabe K, Motohiro M, Sugiura T, Iwasaka T (2009) Prevention of contrast-induced nephropathy by chronic pravastatin treatment in patients with cardiovascular disease and renal insufficiency. Journal of cardiology 54 (2):192-198. doi:10.1016/j.jjcc.2009.05.006

98. Budano C, Levis M, D'Amico M, Usmiani T, Fava A, Sbarra P, Burdese M, Segoloni GP, Colombo A, Marra S (2011) Impact of contrast-induced acute kidney injury definition on clinical outcomes. American heart journal 161 (5):963-971. doi:10.1016/j.ahj.2011.02.004

99. Balli M, Tasolar H, Cetin M, Hatem E, Cagliyan CE, Seker T, Cayli M (2016) Is atrial fibrillation a risk factor for contrast-induced nephropathy in patients with ST-elevation myocardial infarction? Journal of cardiology 67 (4):327-330. doi:10.1016/j.jjcc.2015.09.018

100. Barbieri L, Verdoia M, Schaffer A, Cassetti E, Marino P, Suryapranata H, De Luca G, Novara Atherosclerosis Study G (2015) Uric acid levels and the risk of Contrast Induced Nephropathy in patients undergoing coronary angiography or PCI. Nutr Metab Cardiovasc Dis 25 (2):181-186. doi:10.1016/j.numecd.2014.08.008

101. Capodanno D, Ministeri M, Dipasqua F, Dalessandro V, Cumbo S, Gargiulo G, Tamburino C (2016) Risk prediction of contrast-induced nephropathy by ACEF score in patients undergoing coronary catheterization. Journal of cardiovascular medicine (Hagerstown, Md) 17 (7):524-529. doi:10.2459/JCM.0000000000000215

102. Demircelik MB, Kurtul A, Ocek H, Cakmak M, Ureyen C, Eryonucu B (2015) Association between Platelet-to-Lymphocyte Ratio and Contrast-Induced Nephropathy in Patients Undergoing Percutaneous Coronary Intervention for Acute Coronary Syndrome. Cardiorenal medicine 5 (2):96-104. doi:10.1159/000371496

103. Kolte D, Spence N, Puthawala M, Hyder O, Tuohy CP, Davidson CB, Sheldon MW, Laskey WK, Abbott JD (2016) Association of radial versus femoral access with contrast-induced acute kidney injury in patients undergoing primary percutaneous coronary intervention for ST-elevation myocardial infarction. Cardiovascular revascularization medicine : including molecular interventions 17 (8):546-551. doi:10.1016/j.carrev.2016.07.008

104. Lazaros G, Zografos T, Oikonomou E, Siasos G, Georgiopoulos G, Vavuranakis M, Antonopoulos A, Kalogeras K, Tsalamandris S, Tousoulis D (2016) Usefulness of C-Reactive Protein as a Predictor of Contrast-Induced Nephropathy After Percutaneous Coronary Interventions in Patients With Acute Myocardial Infarction and Presentation of a New Risk Score (Athens CIN Score). The American journal of cardiology 118 (9):1329-1333. doi:10.1016/j.amjcard.2016.07.069

105. Li Y, Liu Y, Shi D, Yang L, Liang J, Zhou Y (2016) Insulin Resistance Increases the Risk of Contrast-Induced Nephropathy in Patients Undergoing Elective Coronary Intervention. Angiology 67 (2):139-145. doi:10.1177/0003319715578997

106. Mizuno A, Ohde S, Nishizaki Y, Komatsu Y, Niwa K (2015) Additional value of the red blood cell distribution width to the Mehran risk score for predicting contrast-induced acute kidney injury in patients with ST-elevation acute myocardial infarction. Journal of cardiology 66 (1):41-45. doi:10.1016/j.jjcc.2014.09.006

107. Ozkaynak B, Kayalar N, Gumus F, Yucel C, Mert B, Boyacioglu K, Erentug V (2014) Time from cardiac catheterization to cardiac surgery: a risk factor for acute kidney injury? Interact Cardiovasc Thorac Surg 18 (6):706-711. doi:10.1093/icvts/ivu023

108. Sany D, Refaat H, Elshahawy Y, Mohab A, Ezzat H (2014) Frequency and risk factors of contrast-induced nephropathy after cardiac catheterization in type II diabetic patients: a study among Egyptian patients. Renal failure 36 (2):191-197. doi:10.3109/0886022X.2013.843400

109. Liwen Bao JB, Bo Jin, Nanqing Xiong, Huanchun Ni, Jian Li, Xinping Luo, Haiming Shi, Jinjin Zhang (2018) Clinical Features of CIN in ChineseVery Elderly Patients Undergoing Coronary Angiography Procedure With Hydration Treatment: A Three-Center, Prospective Study. International Journal of Gerontology 12:196-199

110. Kul S, Uyarel H, Kucukdagli OT, Turfan M, Vatankulu MA, Tasal A, Erdogan E, Asoglu E, Sahin M, Guvenc TS, Goktekin O (2015) Zwolle risk score predicts contrast-induced acute kidney injury in STEMI patients undergoing PCI. Herz 40 (1):109-115. doi:10.1007/s00059-013-3957-1

111. Pancholy MS, Skelding K, Scott T, Blankenship J, Pancholy SB (2017) Effect of Access Site Choice on Acute Kidney Injury After Percutaneous Coronary Intervention. The American journal of cardiology 120 (12):2141-2145. doi:10.1016/j.amjcard.2017.08.039

112. Shin DH, Choi DJ, Youn TJ, Yoon CH, Suh JW, Kim KI, Cho YS, Cho GY, Chae IH, Kim CH (2011) Comparison of contrast-induced nephrotoxicity of iodixanol and iopromide in patients with renal insufficiency undergoing coronary angiography. The American journal of cardiology 108 (2):189-194. doi:10.1016/j.amjcard.2011.03.019

113. Wybraniec MT, Chudek J, Bozentowicz-Wikarek M, Mizia-Stec K (2017) Prediction of contrast-induced acute kidney injury by early post-procedural analysis of urinary biomarkers and intra-renal Doppler flow indices in patients undergoing coronary angiography. Journal of interventional cardiology 30 (5):465-472. doi:10.1111/joic.12404

114. Aguiar-Souto P, Ferrante G, Del Furia F, Barlis P, Khurana R, Di Mario C (2010) Frequency and predictors of contrast-induced nephropathy after angioplasty for chronic total occlusions. International journal of cardiology 139 (1):68-74. doi:10.1016/j.ijcard.2008.10.006

115. Marenzi G, De Metrio M, Rubino M, Lauri G, Cavallero A, Assanelli E, Grazi M, Moltrasio M, Marana I, Campodonico J, Discacciati A, Veglia F, Bartorelli AL (2010) Acute hyperglycemia and contrast-induced nephropathy in primary percutaneous coronary intervention. American heart journal 160 (6):1170-1177. doi:10.1016/j.ahj.2010.09.022

116. Pakfetrat M, Nikoo MH, Malekmakan L, Tabande M, Roozbeh J, Ganbar Ali RJ, Khajehdehi P (2010) Comparison of risk factors for contrast-induced acute kidney injury between patients with and without diabetes. Hemodial Int 14 (4):387-392. doi:10.1111/j.1542-4758.2010.00469.x

117. Sgura FA, Bertelli L, Monopoli D, Leuzzi C, Guerri E, Sparta I, Politi L, Aprile A, Amato A, Rossi R, Biondi-Zoccai G, Sangiorgi GM, Modena MG (2010) Mehran contrast-induced nephropathy risk score predicts short- and long-term clinical outcomes in patients with ST-elevation-myocardial infarction. Circulation Cardiovascular interventions 3 (5):491-498. doi:10.1161/CIRCINTERVENTIONS.110.955310

118. Stolker JM, McCullough PA, Rao S, Inzucchi SE, Spertus JA, Maddox TM, Masoudi FA, Xiao L, Kosiborod M (2010) Pre-procedural glucose levels and the risk for contrast-induced acute kidney injury in patients undergoing coronary angiography. Journal of the American College of Cardiology 55 (14):1433-1440. doi:10.1016/j.jacc.2009.09.072

119. Raposeiras-Roubin S, Abu-Assi E, Ocaranza-Sanchez R, Alvarez-Alvarez B, Cambeiro-Gonzalez C, Fandino-Vaquero R, Garcia-Castelo A, Garcia-Acuna JM, Gonzalez-Juanatey JR (2013) Dosing of iodinated contrast volume: a new simple algorithm to stratify the risk of contrast-induced nephropathy in patients with acute coronary syndrome. Catheterization and cardiovascular interventions : official journal of the Society for Cardiac Angiography & Interventions 82 (6):888-897. doi:10.1002/ccd.24847

120. Aykan AC, Gul I, Gokdeniz T, Kalaycioglu E, Turan T, Boyaci F, Erkan H, Hatem E, Aykan DA, Celik S (2013) Is coronary artery disease complexity valuable in the prediction of contrast induced nephropathy besides Mehran risk score, in patients with ST elevation myocardial infarction treated with primary percutaneous coronary intervention? Heart, lung & circulation 22 (10):836-843. doi:10.1016/j.hlc.2013.03.085

121. Koo HM, Doh FM, Ko KI, Kim CH, Lee MJ, Oh HJ, Han SH, Kim BS, Yoo TH, Kang SW, Choi KH (2013) Diastolic dysfunction is associated with an increased risk of contrast-induced nephropathy: a retrospective cohort study. BMC nephrology 14:146. doi:10.1186/1471-2369-14-146

122. Aoki T, Ishii H, Tanaka A, Suzuki S, Ichimiya S, Kanashiro M, Murohara T (2019) Influence of chronic kidney disease and worsening renal function on clinical outcomes in patients undergoing primary percutaneous coronary intervention. Clinical and experimental nephrology 23 (2):182-188. doi:10.1007/s10157-018-1622-y

123. Arbel Y, Fuster V, Baber U, Hamza TH, Siami FS, Farkouh ME (2019) Incidence, determinants and impact of acute kidney injury in patients with diabetes mellitus and multivessel disease undergoing coronary revascularization: Results from the FREEDOM trial. International journal of cardiology 293:197-202. doi:10.1016/j.ijcard.2019.05.064

124. Azzalini L, Poletti E, Lombardo F, Laricchia A, Beneduce A, Moscardelli S, Bellini B, Maccagni D, Cappelletti A, Ancona MB, Carlino M, Chieffo A, Colombo A, Montorfano M (2019) Risk of contrast-induced nephropathy in patients undergoing complex percutaneous coronary intervention. International journal of cardiology 290:59-63. doi:10.1016/j.ijcard.2019.04.043

125. Cinar T, Tanik VO, Arugaslan E, Karabag Y, Cagdas M, Rencuzogullari I, Keskin M (2019) The association of PRECISE-DAPT score with development of contrast-induced nephropathy in patients with ST-elevation myocardial infarction undergoing primary percutaneous coronary intervention. Cardiovascular intervention and therapeutics 34 (3):207-215. doi:10.1007/s12928-018-0545-6

126. Gungoren F, Besli F, Tanriverdi Z, Demirbag R (2018) Inferior vena cava assessment can predict contrast-induced nephropathy in patients undergoing cardiac catheterization: A single-center prospective study. Echocardiography 35 (12):1915-1921. doi:10.1111/echo.14157

127. Matejka J, Varvarovsky I, Rozsival V, Herman A, Blaha K, Vecera J, Lazarak T, Novotny V, Muzakova V, Vojtisek P (2016) Heart failure is the strongest predictor of acute kidney injury in patients undergoing primary percutaneous coronary intervention for ST-elevation myocardial infarction. Kardiologia polska 74 (1):18-24. doi:10.5603/KP.a2015.0115

128. Otsuka K, Shimada K, Katayama H, Nakamura H, Ishikawa H, Takeda H, Fujimoto K, Kasayuki N, Yoshiyama M (2019) Prognostic significance of renal dysfunction and its change pattern on outcomes in patients with acute coronary syndrome treated with emergent percutaneous coronary intervention. Heart and vessels 34 (5):735-744. doi:10.1007/s00380-018-1291-5

129. Schmucker J, Fach A, Becker M, Seide S, Bunger S, Zabrocki R, Fiehn E, Wurmann-Busch B, Pohlabeln H, Gunther K, Ahrens W, Hambrecht R, Wienbergen H (2018) Predictors of acute kidney injury in patients admitted with ST-elevation myocardial infarction - results from the Bremen STEMI-Registry. Eur Heart J Acute Cardiovasc Care 7 (8):710-722. doi:10.1177/2048872617708975

130. Sinkovic A, Masnik K, Mihevc M (2019) Predictors of acute kidney injury (AKI) in high-risk ST-elevation myocardial infarction (STEMI) patients: A single-center retrospective observational study. Bosn J Basic Med Sci 19 (1):101-108. doi:10.17305/bjbms.2018.3797

131. Yildiz I, Yildiz PO, Rencuzogullari I, Karabag Y, Cagdas M, Burak C, Gurevin MS (2019) Association of Serum Osmolarity With Contrast-Induced Nephropathy in Patients With ST-Segment Elevation Myocardial Infarction. Angiology 70 (7):627-632. doi:10.1177/0003319719826466

132. Wang Y, Zhao HW, Zhang XJ, Chen BJ, Yu GN, Hou AJ, Luan B (2019) CHA2DS2-VASC score as a preprocedural predictor of contrast-induced nephropathy among patients with chronic total occlusion undergoing percutaneous coronary intervention: a single-center experience. BMC cardiovascular disorders 19 (1):74. doi:10.1186/s12872-019-1060-0

133. Uzunhasan I, Yildiz A, Arslan S, Abaci O, Kocas C, Kocas BB, Cetinkal G, Dalgic Y, Karaca OS, Dogan SM (2017) Contrast-Induced Acute Kidney Injury Is Associated With Long-Term Adverse Events in Patients With Acute Coronary syndrome. Angiology 68 (7):621-626. doi:10.1177/0003319716676173

134. Gurm HS, Dixon SR, Smith DE, Share D, Lalonde T, Greenbaum A, Moscucci M, Registry BMC (2011) Renal function-based contrast dosing to define safe limits of radiographic contrast media in patients undergoing percutaneous coronary interventions. Journal of the American College of Cardiology 58 (9):907-914. doi:10.1016/j.jacc.2011.05.023
